# Altered morphology and diffusivity of water confined in MXenes: Machine learning–accelerated computations combined with experiments

**DOI:** 10.1126/sciadv.adz1780

**Published:** 2026-03-25

**Authors:** Jiawei Tang, Weiwei Sun, Chaofan Chen, Lars Bannenberg, Xuehang Wang, Tingwei Zhu, Litao Sun, Jinlan Wang, Guobing Ying, Yu Xie, Naresh C. Osti, Alexander I. Kolesnikov, Eugene Mamontov, Madhusudan Tyagi, Jingsong Huang, Paul R. C. Kent

**Affiliations:** ^1^ School of Electronic Science and Engineering, Southeast University, Nanjing 210096, China.; ^2^ Key Laboratory of Quantum Materials and Devices of Ministry of Education, School of Physics, Southeast University, Nanjing 211189, China.; ^3^ Department of Radiation Science and Technology, Faculty of Applied Sciences, Delft University of Technology, Delft, 2629JB, Netherlands.; ^4^ School of Materials Science and Engineering, Southeast University, Nanjing 211189, China.; ^5^ Key Laboratory of Material Simulation Methods and Software of Ministry of Education, College of Physics, Jilin University, Changchun 130012, China.; ^6^ Neutron Scattering Division, Oak Ridge National Laboratory, Oak Ridge, TN 37831, USA.; ^7^ NIST Center for Neutron Research, National Institute of Standards and Technology, Gaithersburg, MD 20899, USA.; ^8^ Department of Materials Science, University of Maryland, College Park, MD 20742, USA.; ^9^ Center for Nanophase Materials Sciences, Oak Ridge National Laboratory, Oak Ridge, TN 37831, USA.; ^10^ Computational Sciences and Engineering Division, Oak Ridge National Laboratory, Oak Ridge, TN 37831, USA.

## Abstract

Nanoconfined water exhibits unique properties compared to bulk water due to limited quantities, frustrated hydrogen bonding, and surface interactions, which are fundamental for energy storage and transport applications. We integrate machine learning–accelerated ab initio molecular dynamics with x-ray diffraction (XRD) and inelastic neutron scattering (INS) to systematically analyze the thermodynamic and dynamic behavior of water confined between functionalized (-F, -O, and -OH) two-dimensional (2D) Ti_3_C_2_T*_x_* MXene layers. As water intercalates between layers, the interlayer spacing exhibits layer-dependent staging characteristics. The water polarization can be flipped by the count and morphology of intercalated molecules interacting with MXene surface groups, resulting in varying electrostatic potential profiles. On the basis of interfacial electrostatic potential, hydrogen bond lifetime, and molecular orientation, we establish a linear combination of exponential model describing water diffusivity. These computational insights align well with experimental x-ray and neutron measurements, suggesting strategies for tuning water morphology and transport by tailoring MXene surface chemistry and water content for electrochemical energy storage and nanofluidic applications.

## Teaser

Machine learning-accelerated ab initio molecular dynamics address MXene-water interfaces to inform a diffusion model.

## INTRODUCTION

Water, the most ubiquitous and indispensable substance for humans and other organisms, plays a crucial role in various fundamental biological and chemical processes. The confinement of water within cavities or on surfaces at the nanoscale is prevalent in natural systems, resulting in unique physical properties compared to those of bulk water (*1*, *2*). One intriguing aspect of investigating water under nanoconfinement is unraveling its anomalous behavior and the unknown physics and chemistry behind the apparent phenomenon, such as ultrafast flow (*3*), new structure and phase transitions (*4*, *5*), and exceptionally low dielectric constant (*6*). Moreover, the unique states of water at the solid-liquid interfaces (SLIs), defined by the structural, thermodynamic, and dynamic characteristics of water near the interface (*7*), establish the foundation for a spectrum of technological domains. These include, but are not limited to, electrochemical energy conversion and storage systems (*8*), hydrovoltaic power generation (*9*), sensor technologies (*10*), and corrosion prevention strategies (*11*).

The variable properties of solid substrates assembling the SLI, including geometry, composition, and surface characteristics, can considerably alter water behaviors under confinement, offering opportunities for water manipulation. Particularly in layered two-dimensional (2D) materials, where water molecules are trapped between adjacent sheets, the degree of confinement and interfacial chemistry together govern water’s configuration, mobility, and interaction with charge carriers (*12*, *13*). Extensive research has revealed the peculiar transport behavior of water confined within carbon-based materials, ranging from carbon nanotubes (CNTs), graphene nanochannels (*14*–*16*), to graphene oxide sheets (*17*). Transition-metal dichalcogenides and hexagonal boron nitride (hBN) have been shown to facilitate water flux through reducing hydraulic resistance (*18*, *19*). MXenes, a family of 2D transition-metal carbides, nitrides, and carbonitrides, offer unprecedented versatility of surface groups, from conventional terminations such as T*_x_* = -F, -O, and -OH (*20*) to recently synthesized -Se, -Te, -Cl, and -Br via the molten salt method (*21*–*23*). This wide range of surface chemistries renders MXenes an ideal playground for altering the behaviors of water confined with various types of SLIs.

Regulating the state of water confined between MXene layers has emerged as a foundational strategy for controlling charge storage kinetics and electric-double layer (EDL) formation at electrochemical interfaces across diverse aqueous electrolytes (*24*–*26*). Particularly for Ti_3_C_2_T*_x_*, the first reported and most studied MXene, its high electrical conductivity (up to 20,000 S cm^−1^) and hydrophilicity enable excellent electrochemical energy storage performance, such as high capacitance and great cycling stability (*27*–*29*). Enhancing the nanoconfined water amount and mobility could largely boost the proton diffusivity and accessibility, thereby improving the power density and charge storage capacity (*30*, *31*). The compact and well-organized water layers facilitate the establishment of an interconnected hydrogen bond (HB) network for rapid proton transfer (*32*). Beyond proton redox activity in pseudocapacitors, confined water also critically governs the hydration structure of metal ions within the EDL (*33*, *34*). Gao *et al.* (*35*) emphasized that the capacitance of Ti_3_C_2_T*_x_* MXenes is notably influenced by the number of hydrated water molecules and the position of the hydrated ion through a blend of experimental and theoretical investigations. Hence, understanding the role of water at SLIs, particularly under confinement of Ti_3_C_2_T*_x_*, is profound for unlocking further improvements in supercapacitor performance, but it remains elusive due to the complexity and somewhat undetectable feature at atomic scale. Moreover, manipulating water molecules by the surface group and unraveling the responsive mechanism to the MXene surface remain challenging, necessitating a comprehensive understanding of SLI through sophisticated and in-depth investigations.

Elucidating the atomic-scale structure and properties at SLIs, either through direct characterizations or theoretical approaches, offers a valuable opportunity to advance our understanding. This includes tracking water molecule intercalations, understanding the evolution of interlayer spacings, analyzing water polarization and diffusion, etc. Shpigel *et al.* (*36*) quantitatively distinguished between the water trapped in the Ti_3_C_2_T*_x_* interlayer spacings and that within the mesoporous volume by using advanced in situ hydrodynamic spectroscopy. Neutron scattering enabled the dynamic analysis of both weakly and strongly confined water while simultaneously resolving the bonding between Ti_3_C_2_T*_x_* MXene surfaces and hydrogenous molecules (*37*, *38*). In situ x-ray diffraction (XRD) revealed fluctuating expansion and shrinkage of Ti_3_C_2_T*_x_* during the charging and discharging in a sulfuric acid electrolyte, highlighting the key role of interlayer spacing and water intercalation (*26*). Despite these experimental advances, complementary theoretical modeling is indispensable in capturing the full complexity of SLIs. Traditional ab initio molecular dynamics (AIMD) methods can offer detailed insights into the atomic-level interactions and dynamics of water molecules but are computationally intensive and scale limited. Hou *et al.* (*39*) applied the deep learning potential molecular dynamics to investigate the water diffusivity in the monolayer (ML) form under different proton concentrations confined between V_2_CT*_x_* (T = -O and -OH) MXene layers. The varying concentrations of protons can result in an adjustable oxidation rate, the formation of a hexagonal ice phase at ambient temperature, and a notable reduction in water diffusivity. The highly correlated descriptors and clear mechanisms that underlie these phenomena remain elusive, emphasizing the necessity for a comprehensive understanding of the interactions between MXene and water, along with the factors influencing water diffusivity. The advent of the on-the-fly machine learning force field (MLFF) techniques (*40*) enables far more rapid and large-scale molecular dynamics modeling with near ab initio accuracy without preparing a massive training dataset, performing rounds of training process and validation, which might be a more suitable strategy to deeply investigate the MXene-water interface and associated thermodynamic and dynamic changes.

Here, we establish a multimodal investigation framework combining density functional theory (DFT), machine learning–accelerated molecular dynamics (MLaMD), and versatile experimental probes [including XRD, inelastic neutron scattering (INS), and zeta potential measurements] to systematically decode the surface-dependent properties of water confined in Ti_3_C_2_T_2_ (T = -F, -O, and -OH) MXenes. Here, T_2_ refers to the calculated models with a single termination, in contrast to T*_x_* for experimentally synthesized MXenes with mixed terminations. In particular, we show that MXene surface terminations and intercalated water loading jointly sculpt the local electrostatic potential, which, in turn, dictates HB network connectivity and water orientation. By correlating termination-dependent electrostatic landscapes with water morphology, HB dynamics, and diffusion coefficients, we uncover key descriptors that govern water mobility under nanoconfinement in an exponential manner. Notably, electroactive water species can modulate the self-diffusion coefficient by over an order of magnitude, profoundly affecting mass transport in high-rate supercapacitors. Our study casts light on the fundamental mechanisms of water diffusion within MXene layers, enhancing our comprehension of MXene-based interfacial architectures and providing actionable guidance for improving the performance of energy storage devices.

## RESULTS

### Surface-dependent water intercalation and structural response

**Fig. 1.**
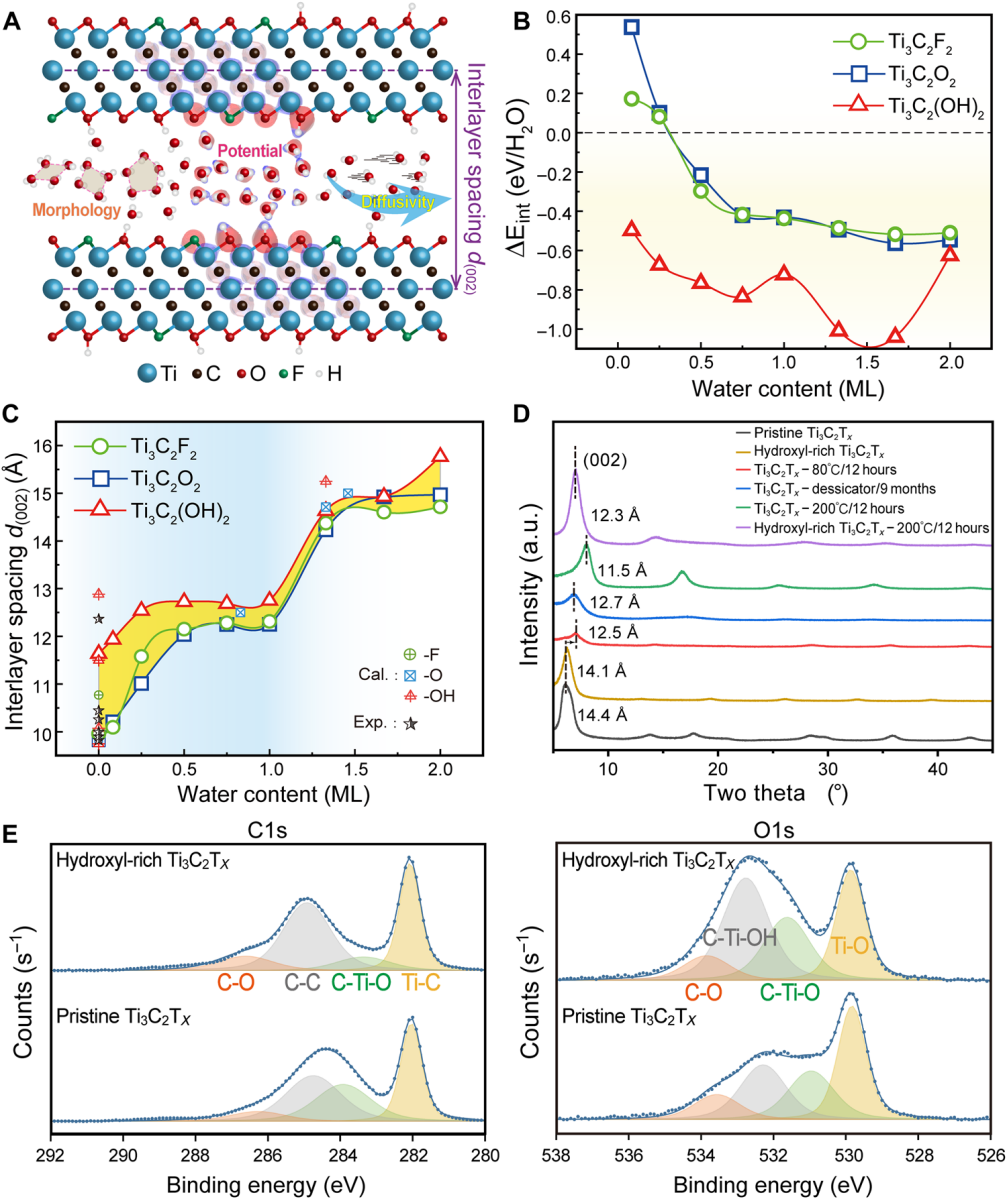
Evolutions of the water intercalation–related energy and the *d*-spacing. (**A**) Schematic illustration of the water confined between MXene sheets and the resulting changes in morphology, potential, and diffusivity. (**B**) Intercalation energy per H_2_O as a function of the water content. (**C**) Change of interlayer spacing *d*_(002)_ upon water insertion into Ti_3_C_2_T_2_ MXenes. The calculated and experimental values from (*20*, *43*–*48*), identified as Cal. and Exp., are presented for comparison. (**D**) XRD patterns of pristine, hydroxyl-rich, and variously dehydrated Ti_3_C_2_T*_x_*. (**E**) High-resolution C1s and O1s XPS spectra of pristine and hydroxyl-rich Ti_3_C_2_T*_x_*. a.u., arbitrary units.

The MXene-water SLI constitutes a complex system characterized by intricate interactions between the MXene surface and water molecules, as well as among water molecules themselves. Such interdependent interactions establish a unique environment that is decisive to both the static and dynamic signatures of the confined water. The tight arrangement of water molecules is found to facilitate the assembly of well-aligned, isotropic MXene-bridged graphene nanoplatelets (*41*). As shown in Fig. 1A, surface groups of MXenes in direct contact with water can restructure the morphology of the water layer in response to hydration levels, thereby affecting the electrostatic potential landscape and the diffusivity of water molecules. Before delving into the nanoscale dynamics of confined water, we first examine the energetics driving water intercalation with different surface groups.

Here, the confined water content is found to affect the insertion energetics, as reflected by the intercalation energy per water molecule versus water content (Fig. 1B), expressed as

$${\Delta E}_{\text{int}}=\frac{E_{\text{tot}}\left({\text{Ti}}_{3}C_{2}T_{2}\cdot xH_{2}O\right)-E_{\text{tot}}\left({\text{Ti}}_{3}C_{2}T_{2}\right)-x\cdot E_{\text{tot}}\left(H_{2}O\right)}{x}$$ (1)

where $E_{\text{tot}}\left({\text{Ti}}_{3}C_{2}T_{2}\cdot xH_{2}O\right)$ is the total energy of the hydrated MXene system incorporating *x* water molecules, $E_{\text{tot}}\left({\text{Ti}}_{3}C_{2}T_{2}\right)$ is the energy of dry Ti_3_C_2_T_2_ bulk, and $x\cdot E_{\text{tot}}\left(H_{2}O\right)$ is the energy sum of *x* isolated water molecules. By definition, a positive (negative) value would indicate an endothermic (exothermic) process for the intercalation, respectively. The evolution of the intercalation energy of water in Ti_3_C_2_F_2_ and Ti_3_C_2_O_2_ demonstrates consistency, as shown in Fig. 1B. That is, the intercalation energy ${\Delta E}_{\text{int}}$ is positive below 0.31 ML and then experiences a dip and reaches a plateau around ~−0.5 eV/H_2_O as the intercalated water increases. In contrast, the -OH terminated interface remains negative ${\Delta E}_{\text{int}}$ across the full range and stands below the -F and -O terminated ones with a sizeable gap, demonstrating its stronger energetic favorability.

Notably, the -OH system exhibits an anomalous elevation in ${\Delta E}_{\text{int}}$ at 1 ML, associated with lattice distortion and water layer reorganization, and another increase at 2 ML, primarily due to weakened interfacial coupling (details in table S1 and fig. S3). This energetic preference, however, diminishes at higher water content exceeding 2 ML, because once the hydration layer is completed, subsequent water behaves nearly bulk-like, and ${\Delta E}_{\text{int}}$ approaches the cohesive energy of bulk water (−0.57 eV/H_2_O), with only minor oscillations from discrete layering. Moreover, the entropy change (∆S) of confined water is also estimated and presented in fig. S4A. The entropic contribution (T∆S) is nearly constant and about one order of magnitude smaller than the corresponding ${\Delta E}_{\text{int}}$ (fig. S4, B to D), confirming that entropy has only a minor influence on the free-energy change and does not affect the intercalation behavior. Consistent with this, Vaitheeswaran *et al.* (*42*) reported that water intercalation in CNTs is primarily energy driven rather than entropy driven. The present theoretical results robustly supports the experimental observation that an optimal proportion of -OH terminations facilitates the initial intercalation of water molecules (*32*). Overall, via elucidating intercalation energetics, we provide fundamental insights into the role of surface functional groups in regulating water filling within the MXene interlayer channels.

Among all structural parameters, the interlayer spacing [i.e., *d*-spacing of (002) facet, *d*_(002)_, in Fig. 1A] of MXenes is an observable and crucial parameter that is related to the water intercalation and can profoundly affect electrochemical behavior (*31*). Our calculations reveal that the interlayer spacing exhibits a two-stage characteristic enhancement with increasing intercalated water (Fig. 1C). These results agree excellently with prior measurements (*20*, *43*–*48*), particularly those by Ghidiu *et al.* (*49*), who identified interlayer spacings of 12.5 and 15 Å corresponding to ML and bilayer water configurations, respectively, thereby confirming the validity and reliability of our models. Most of the experimental interlayer spacings of anhydrous MXenes fall into the theoretical range between 9.8 and 11.6 Å bounded by the three terminated (-F, -O, and -OH) structures, in line with the nature of mixed surface groups in reality, and the spacing of Ti_3_C_2_(OH)_2_ is the largest one of the three surfaces. When the intercalated water is above 1 ML, the interlayer spacing becomes less sensitive to the surface groups. Pure termination models act as bounding cases that encapsulate the essential structural response to surface terminations and hydration levels, showing semiquantitative agreement with experimental result.

With the calculated interlayer spacing and corresponding intercalated water content for Ti_3_C_2_T_2_, one could compare with the experimentally measured *c*-lattice parameters. For instance, the Ti_3_C_2_T*_x_* immersed in water and sulfuric acid before electrochemical cycling arrived at approximately *c*/2 = 12.8 to 13.9 Å (*32*, *43*), corresponding to about 1 to 1.28 ML water intercalated into MXene. Our independent measurements presented in Fig. 1D show that the fresh Ti_3_C_2_T*_x_* exhibits a layer spacing of ~14.4 Å, corroborating the assignment of 1.33 ML water. Following overnight vacuum drying at 80°C or storage in a desiccator under vacuum for 9 months, the pristine Ti_3_C_2_T*_x_* attained partially dehydrated states evidenced by contraction of layer spacing to 12.5 and 12.7 Å, respectively, corresponding to 0.25 to 1 ML interlayer water. Further annealing Ti_3_C_2_T*_x_* at 200°C in vacuum for 12 hours induced almost complete dehydration and a shrinkage of *d*-spacing to 11.5 Å (<0.25 ML water). Overall, the interlayer spacing parameters calculated on the basis of pure surface groups reasonably frame the experimental values observed in synthesized MXenes, providing a quantitative reference for deducing the confined water content from lattice changes.

In experiments, the ratio among different surface groups of Ti_3_C_2_T*_x_* MXene can be tuned electrochemically (*43*, *50*). We reduced the Ti_3_C_2_T*_x_* in 1 M H_2_SO_4_ by applying a constant voltage of −0.8 V versus Ag for 1 hour. The as-obtained hydroxyl-rich Ti_3_C_2_T*_x_* was washed with deionized (DI) water to remove the electrolytes and dried at 200°C in vacuum for 12 hours before the XRD measurement. As shown in Fig. 1E, x-ray photoelectron spectroscopy (XPS) shows that more -OH groups are introduced onto MXene surfaces while maintaining the same amount of -F groups, changing the -OH/-O ratio from 1:1 to 1:0.62. The complete XPS survey scan and the corresponding element composition analysis are provided in fig. S5 and table S2. The hydroxyl-rich Ti_3_C_2_T*_x_* after vacuum annealing exhibits an expanded *d*-spacing of 12.3 Å, larger than that of pristine MXene (11.5 Å), mirroring the trend in the calculated results, shown in Fig. 1C.

To better understand the effect of water removal on interlayer spacing, we performed in situ XRD on MXene films while gradually increasing the temperature in 50°C increments (fig. S6). As the temperature rises from 25° to 150°C, the (002) diffraction peak shifts rightward and broadened, indicating a progressive reduction in interlayer spacing and increased structural disorder. A similar trend was observed in the (004) peak. This contraction in *d*-spacing corresponds to the loss of physically adsorbed water, aligning with nuclear magnetic resonance studies that showed an evident decrease in free water content after annealing at 200°C (*51*). Likewise, thermogravimetric analysis confirmed water loss below 200°C (*52*, *53*). These findings are consistent with simulation results, which also predict interlayer shrinkage upon water removal. At temperatures exceeding 200°C, -OH groups become less stable and begin to detach from the MXene surface, leading to the appearance of a new diffraction peak at 2θ = 7.8°, which remained unchanged up to 450°C. This thermal fluctuation is also reflected in the calculated deintercalation energies ${\Delta E}_{\text{de}-\text{int}}$ (fig. S7), where -OH terminated systems exhibit more pronounced temperature-dependent variations, although the energy cost remains higher than in -F and -O terminated systems. Notably, the new peak corresponds to a reduced interlayer spacing of 11.3 Å, in agreement with simulations demonstrating that the loss of -OH groups further decreases the *d*-spacing. The concordance between calculations and measurements in the *d*-spacing related responses to changes in surface groups and hydration supports the use of our chosen computational models and methodology to gain insights into the behavior of MXenes and intercalated water in varied conditions.

### Altered water distributions, packings, and orderings

**Fig. 2.**
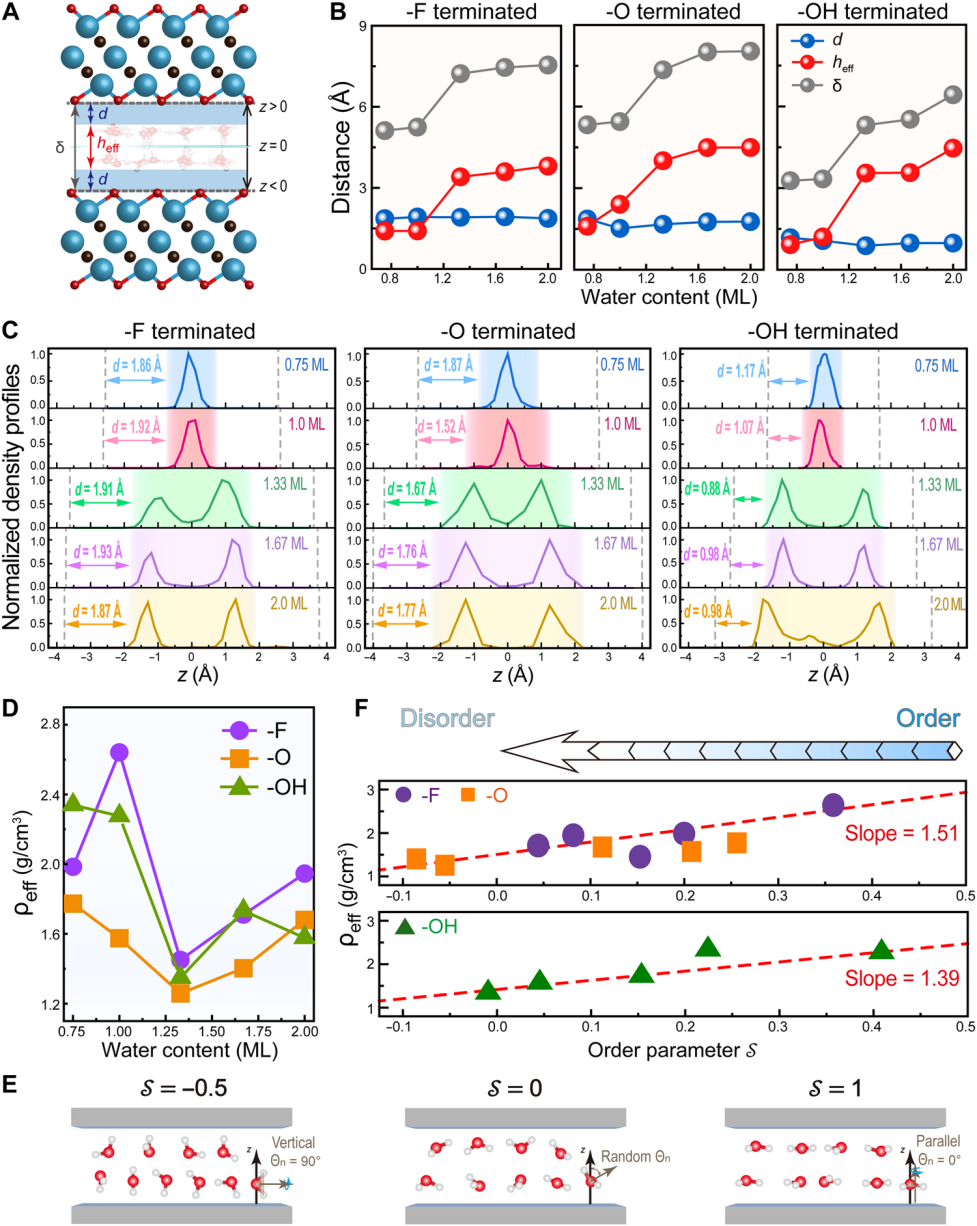
SLI-related structural characterization, water distribution, density, and ordering. (**A**) Cross-sectional view of MXenes with water molecule intercalation: the illustration of exclusion-volume thickness *d*, the effective height $h_{\text{eff}}$, and the confinement distance δ, with the baseline *z* = 0 denoting the central plane of confinement channels. (**B**) The confinement distance δ, the exclusion-volume thickness *d*, and the effective height $h_{\text{eff}}$ as a function of the water content. (**C**) The normalized density distribution of confined water in Ti_3_C_2_T_2_ MXenes. The dotted lines indicate the solid-liquid contacting plane, and the shaded areas indicate the $h_{\text{eff}}$. (**D**) Effective density $\rho_{\text{eff}}$ as a function of the water content. (**E**) Schematic diagram illustrating the relationship between the orientation order parameter 𝒮 and the water morphology. (**F**) Effective density changes induced by the order parameters 𝒮.

The observable structural response of MXene upon water intercalation and the associated thermodynamics have been addressed and compared with different surface groups, both theoretically and experimentally. We next explore the atomic-scale distribution of water molecules, solid-liquid separation, and order morphology, factors that are nearly impossible to measure directly. As delineated in Fig. 2A, the confinement distance δ, acting as a spectator to the interlayer expansion, is composed of the thickness *d* of the exclusion volume and the effective height $h_{\text{eff}}$ of the water layer. The water distribution can also be classified into the upper (*z* > 0) or lower bound (*z* < 0) (Fig. 2A). This decomposition of δ into *d* and $h_{\text{eff}}$, as summarized in Fig. 2B, enables us to explore the physical origin of interlayer expansion.

The MXene-water exclusion thickness (*d*) remains nearly constant across varying levels of intercalated water but follows a termination-dependent trend: *d*_-F_ ≥ *d*_-O_ > *d*_-OH_, as Fig. 2B shows. This trend reflects that the interfacial separation between MXenes and intercalated water is decided by the intrinsic chemical nature of surface groups rather than the water content. In particular, the -OH termination exhibits the strongest hydrophilicity and shortest interfacial HB lengths among the three surface groups, as evidenced in fig. S8, together leading toward the low thickness *d* of the exclusion volume in -OH terminated systems. Moreover, both δ and $h_{\text{eff}}$ increase synchronously with water content for the three surfaces, confirming that $h_{\text{eff}}$ dominates the overall interlayer expansion. As a metric of confined water compactness, $h_{\text{eff}}$ intrinsically reflects the central role of water-water interactions in determining interlayer spacing and thereby provides a tunable handle for the design and control of confined channels. Recent study has shown that even subtle variations in surface electrostatics can perturb water-water interactions and alter confined water structures (*54*). Accordingly, adjusting $h_{\text{eff}}$ via surface functionalization or external stimuli may open a route to expand the interlayer spacing beyond simply loading water content.

A visualized and quantitative characterization of the distribution of water molecules confined within MXene layers is shown in Fig. 2C, via the normalized density profiles of oxygen atoms of water. The sharp and high peaks illustrate the localized position of water. When more than 1 ML water is intercalated, another distinguishable peaks emerge, indicating a well-defined stratification of water layers. Water molecules in all cases are interfacial and confined between MXene layers, exhibiting either even or uneven distributions depending on water content. Notably, the -OH terminated MXenes exhibit the sharpest peaks and the smallest *d* compared with the -F and -O terminated counterparts, where a marked coalescence of the two peaks is observed. The high hydrophilicity of the -OH terminations and their capacity to form robust HBs with water molecules promote a well-defined, distinct stratification in the density profile. Hence, other than the oxidation state, the affinity of surface groups influences the distribution of intercalated water molecules in MXenes, supported by recent spectroscopic evidence that confined water structures are primarily dictated by interfacial chemistry rather than confinement itself (*55*).

The effective density $\rho_{\text{eff}}$ of water could be determined from the phenomenological compactness described by $h_{\text{eff}}$ as $\rho_{\text{eff}}=\frac{m}{A\cdot h_{\text{eff}}}$, where m is the mass of water molecules and A denotes subvolume area of confined water. As illustrated in Fig. 2D, $\rho_{\text{eff}}$ is much higher for the quasi–single-layer water (0.75 or 1 ML) than for the bilayer water (1.33 to 2 ML), with the minimum $\rho_{\text{eff}}$ occurring at the 1.33 ML water intercalation. For the -F termination, $\rho_{\text{eff}}$ is lower at 0.75 ML owing to the larger effective volume ($A\cdot h_{\text{eff}}$). It increases at 1 ML with nearly constant volume and drops to a minimum at 1.33 ML as the volume expands sharply (fig. S9). Note that the $\rho_{\text{eff}}$ with the -O termination is always lower than that with -F or -OH groups for nearly all water content. This disparity can be attributed to the more negative oxidation state of surface groups, which enhances electrostatic repulsion between water molecules and promotes a dispersed spatial distribution.

Another dimension of describing the water structure is the order parameter 𝒮 (*56*, *57*), with orientational characteristic, following the equation of $𝒮=0.5\langle 3{\text{cos}}^{2}\theta_{n}-1\rangle$, where $\theta_{n}$ is defined as the angle between the *z* axis and the normal axis to the H-O-H plane, resulting in a range of 𝒮 from −0.5 to 1. Hereby, we can provide insight into whether the molecules are aligned parallel (𝒮=1), perpendicular (𝒮=−0.5) to the MXene surface, or in a random arrangement (𝒮=0), as illustrated in Fig. 2E. Through the linear correlation between 𝒮 and $\rho_{\text{eff}}$, as displayed in Fig. 2F, we are able to illustrate a decrease of $\rho_{\text{eff}}$ corresponding to a concurrent reduction of the 𝒮 across all three terminations. In other words, a widespread dispersion allows for a variety of orientations, leading to a disordering and lower density. This interdependence among the spatial distribution, orientational order, and effective density constitutes a closed-loop relationship. Such a relationship is also evident in 2D square ice (*58*), where strong ordering minimizes the spatial occupation and increases the density, in contrast to the tetrahedral ordering of bulk ice, which reduces density (*59*). Compared with the 2D square ice (𝒮=1), the MXene-confined water with an 𝒮 ranging from −0.1 to 0.5 exhibits semi- or full disorder. These quantitative characterizations provide robust corroboration for the reduced structural order during the incipient development of another water layer.

### SLI induced polarization flip and electrostatic potential fluctuation

Having elucidated the solid-liquid interaction and deduced the morphology evolution of both water and MXenes, we now turn to investigate the origin of such phenomena. It is well known that the water molecules are highly polar, and interaction with the tunable MXene surface unavoidably perturbs their spontaneous polarization. Here, we use the *z* component of the local polarization density (LPD), written as $P_{z}\left(z\right)=\rho \left(z\right)\mu \left(z\right)$ (*60*, *61*), where ρ(z) is the average local density of water molecules, $\mu \left(z\right)=|\mu |\langle \text{cos}\theta_{d}\rangle$ is the *z* component of the dipole moment vector, with the magnitude of the dipole moment ∣μ∣=2.27 Debye, and $\langle \text{cos}\theta_{d}\rangle$ denotes the average cosine of the dipole orientations, as a way to monitor out-of-plane polarization changes.

**Fig. 3.**
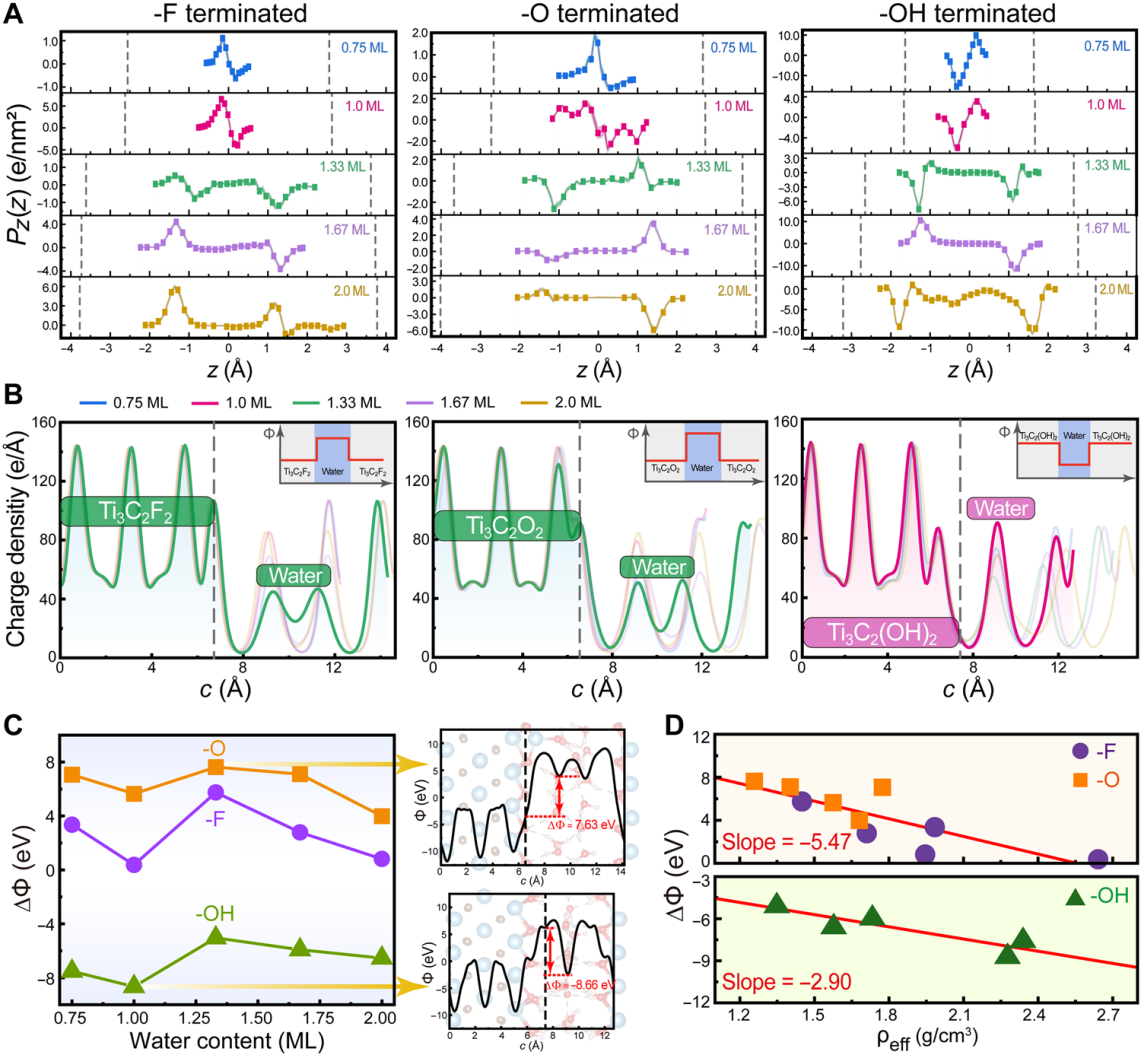
Polarization-induced charge and electrostatic potential distributions at SLIs. (**A**) LPD within confined channels at varying water content. (**B**) 1D planar averaged electronic charge density distributions at Ti_3_C_2_T_2_-water interfaces. Inset: Schematic of electrostatic potential hump or well within the water region. (**C**) Electrostatic potential difference ∆Φ at varying water content. (**D**) Linear relationship between ∆Φ and $\rho_{\text{eff}}$. All the vertical dashed lines indicate the interface position.

Figure 3A shows the LPD profiles, with error bars reported as the SEM estimated from an effective sample size $N_{\text{eff}}$ obtained via integrated autocorrelation time analysis (*62*). The coupling between surface terminations and water loading yields distinct polarization patterns. For both 0.75 and 1 ML water intercalated scenarios, alternative up and down dipole pairs are observed, resulting in an approximately antiparallel polarization. As more water intercalated, particularly beyond 1 ML water, the water stratification effect is present as revealed in Fig. 2C, where the two-peak characteristics near SLI are formed, and the nearly antiparallel polarizations are disrupted with evident and swift flips. The polarizations in the -F terminated case experience a clear flip from 1.33 to 2 ML intercalation. Similarly, disordered polarizations are imposed in the -O terminated case, ultimately presenting an almost unimodal characteristic. Moreover, the water polarizations confined between Ti_3_C_2_(OH)_2_ experience several flips, showcasing a highly responsive behavior to water intercalation. Such flips of water contrast with the antiferroelectric polarization observed in graphene and hBN channels (*63*), emphasizing the intricate dynamics of polarization driven by the MXene-water SLI. Crucially, these polarization flips can redistribute the interfacial charge and reshape the local electric field, directly modulating charge transfer kinetics.

We next analyze the charge transfer induced by the polarized water and the negative clay-like MXene surface, as depicted in Fig. 3B. Specifically, the -F and -O terminations are electron rich, and the water layer is electron poor, thus forming an electrostatic potential hump centered within the water region (Fig. 3B, inset). In contrast, the -OH terminated case presents the opposite picture, i.e., an electrostatic potential well, since the water layer is highly charge accumulated. Notably, the most marked charge density fluctuation is observed with 1.33 ML of water at the -F and -O groups and the 1 ML water layer for the -OH groups. The localized charge density varying with water content reflects the dynamic nature of this interfacial charge transfer, which, in turn, generates the electrostatic potential change.

The observed polarization flips and associated charge density variances at the SLI may generate an internal electrical field (*64*, *65*), which is believed to closely correlate to the electrostatic potential change. Here, we primarily take the SLI-driven electrostatic potential difference ∆Φ, defined as $∆\Phi =\Phi_{W}-\Phi_{M}$, where $\Phi_{W}$ represents the potential of the water layer which maximizes the ΔΦ, and $\Phi_{M}$ signifies the potential of the MXene surface at SLI. Figure 3C quantitatively depicts that the interactions between water molecules and surface groups uniquely alter the electrostatic landscape, with -F and -O terminations creating an electrostatic potential hump (∆Φ>0), in notable contrast to the potential well (∆Φ<0) formed by the -OH groups. The full description of the electrostatic potential for all three surface groups across varying water content can be found in fig. S10. The ∆Φ is a direct consequence of charge redistribution at the molecular level, i.e., the most pronounced electrostatic potential hump or well corresponds to the strongest charge density fluctuations. Moreover, the $\rho_{\text{eff}}$ is negatively correlated with ∆Φ for all three terminations, as demonstrated in Fig. 3D. Briefly, a lower $\rho_{\text{eff}}$ (disordering) boosts the increase of $\Phi_{W}$, resulting in a positive developed ΔΦ. Therefore, $\rho_{\text{eff}}$ serves as an indicator of linking water morphology with ΔΦ, suggesting a potential relationship between structural compactness and electrostatic modulation.

### Hydrogen bonding and morphological and vibrational signatures

The water molecules are connected through HBs, and under confinement and interaction with MXene, their composition, structure, and lifetime will change. In this context, HBs can be classified into three categories based on their environment, e.g., the donor and acceptor moieties: (i) ${\text{HBs}}^{W-W}$ in water, (ii) ${\text{HBs}}^{W-T}$ between water and terminations, and (iii) ${\text{HBs}}^{T-W}$ between terminating groups and water, exclusively applicable to the -OH terminated MXenes. The latter two types are defined as interfacial HBs due to their direct involvement with the MXene surface. For ${\text{HBs}}^{W-W}$, the two- and three-HB characteristics are dominant in 0.75 to 1.33 ML, and the four-HB emerges along with the prevalent three-HB configuration for cases above the 1.33 ML water intercalated, as shown in fig. S11A. The average number of ${\text{HBs}}^{W-W}$ and ${\text{HBs}}^{W-T}$ changes consistently across all three terminations (fig. S11, B and C). While in the case of -OH terminated surface, ${\text{HBs}}^{T-W}$ should be additionally take into account, showing an interesting role on the behavior of the water (fig. S11D). Furthermore, the negative HB interaction energies observed across all the three systems in fig. S12 suggest that hydrogen bonding underpins the interface stability, and the most evident case lies with the -OH terminated ones.

**Fig. 4.**
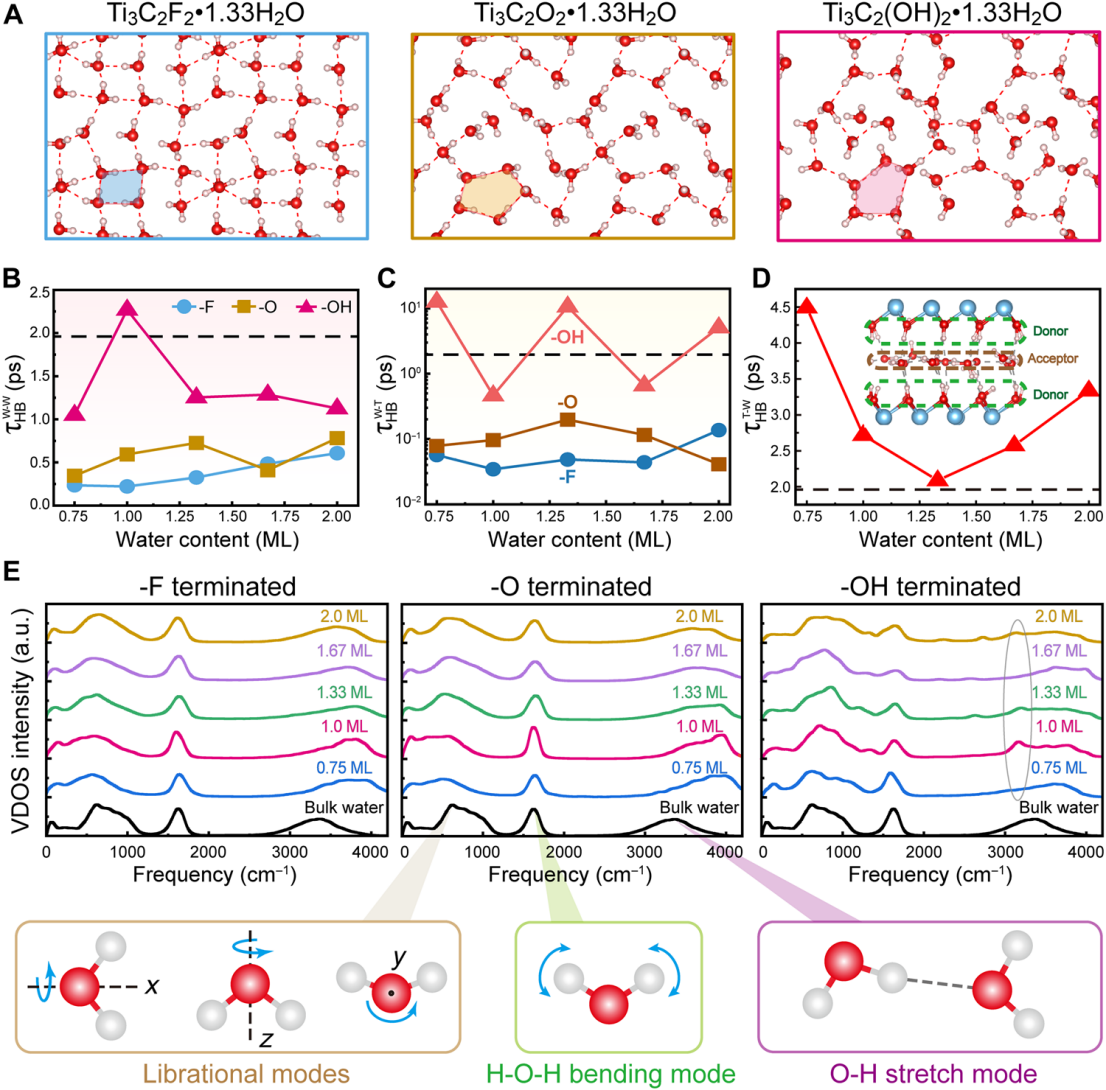
HB dynamics and vibrational properties of confined water. (**A**) Snapshots of HB networks within 1.33 ML water. HBLTs, established (**B**) within confined water $\tau_{\text{HB}}^{W-W}$, (**C**) between confined water (donor) and surface terminations (acceptor) $\tau_{\text{HB}}^{W-T}$, and (**D**) between -OH terminations (donor) and confined water (acceptor) $\tau_{\text{HB}}^{T-W}$. The horizontal dashed lines represent the HBLT of bulk water. (**E**) The vibrational density of states (VDOS) spectra of confined water.

The interplay between polarization and interfacial hydrogen bonding results in the unique HB network within the confined water, as exemplified by the 1.33 ML water shown in Fig. 4A, illustrating the morphological patterns of confined water at SLIs influenced by the surface group. In the case of Ti_3_C_2_F_2_, the bilayer water molecules present a rhombic arrangement, with water molecules forming almost saturated HB networks with neighboring molecules both within the same layer and across the adjacent layer. As the hydrophilicity of surface groups increases, the formation of interfacial HBs between water and the -O and -OH terminations reduces hydrogen bonding within the water layer, leading to the emergence of a hexagonal structure. Moreover, the water morphology at -O and -OH terminations is more disordered compared to that at -F terminations, which is consistent with the above calculated $\rho_{\text{eff}}$ and 𝒮, further highlighting the impact of surface chemistry on the structure and bonding characteristics of confined water. On the basis of our modeling, the confined water experiences a transition from ordered quadrilaterals to disordered pentagons and ultimately to 3D tetrahedral geometries, with increasing water content (fig. S13). This structural evolution is further corroborated by the orientation analysis of HBs, which shows a shift from order to disorder and back to order (fig. S14). The diagram of the water structure transition presented here would stimulate large-scale modeling to depict a more tangible and explicit transition path.

A more consequential, yet often overlooked, aspect is the dynamics of these HBs, informed by the HB lifetime (HBLT), quantified by the continuous HB correlation function C(t) (*66*). The decaying function  C(t) can be approximately estimated by a biexponential function

$$C\left(t\right)\approx A\text{exp}\left(-\frac{t}{\tau_{1}}\right)+\left(1-A\right)\text{exp}\left(-\frac{t}{\tau_{2}}\right)$$ (2)

where $\tau_{1}$ and $\tau_{2}$ represent the fast and the slow decay time constant, respectively, together with the relative importance, *A*. More details about C(t) are provided in figs. S15 to S17. Then, the HBLT $\tau_{\text{HB}}$ can be obtained by Eq. 3 (*67*)

$$\tau_{\text{HB}}=\int_{0}^{\infty }\left[C\left(t\right)-\langle C\left(t=\infty \right)\rangle \right]\mathrm{dt}$$ (3)

Figure 4B elaborates upon the HBLT via $\tau_{\text{HB}}$, with a reference of the bulk water, denoted as the dashed lines. Note that the continuous HBLT of bulk water as 1.96 ps, falling into the reported theoretical range between 0.9 and 2.0 ps (*68*–*70*). In comparison, $\tau_{\text{HB}}^{W-W}$ is found to be always below that of bulk water, except for the -OH terminated MXene with 1 ML water. A shorter HBLT than that of bulk water has been reported in CNT-confined water (*71*). $\tau_{\text{HB}}^{W-W}$ at the -OH terminations considerably exceed those at -F and -O terminations, showing strengthened O-H⋯O, with a similar trend observed for $\tau_{\text{HB}}^{W-T}$ in Fig. 4C. Moreover, -OH terminations as donors all exhibit longer lifetimes $\tau_{\text{HB}}^{T-W}$ compared to those in bulk water, as illustrated in Fig. 4D. It is thus concluded that the -OH surface groups offer a higher quantity of interfacial HBs to the confined water, and these bonds exhibit a longevity either comparable to or exceeding that in bulk water.

Another interest would be the vibrational properties of water under confinement. In nature, vibrational modes of liquid water consist of three major bands, (i) the O-H stretch mode at 3000 to 4000 cm^−1^, (ii) H-O-H bending mode at ~1600 cm^−1^, and (iii) librational modes at ~585 cm^−1^ (*72*). Here, we computed the vibrational density of states spectra from the velocity autocorrelation as the spectrum characterization of water dynamics, useful to identify the vibrational signatures of confined water, e.g., new vibration modes. In Fig. 4E, the position of the H-O-H bending mode in confined water is still well aligned with that of bulk water only experiencing subtle variance for all surfaces. However, the peak assigned to the O-H stretch mode has been broadened and blue-shifted, ascribed to the more dispersive water molecules. Notably, in -OH terminated MXenes, particularly with 1 ML water, the spectrum shows a distinct peak at 3200 cm^−1^, implying the presence of four-coordinated H-bonded water, which is strongly associated with the additional ${\text{HBs}}^{T-W}$ and longer HBLTs induced by -OH terminations. The primary role of confinement on the vibrational behavior is lying on the librational mode, an indicator of the integrity of the HB network. For -F and -O terminations, the librational mode of the confined water is red-shifted compared with the bulk water, indicating a softening of the water structure in a confined environment. In essence, surface groups and confinement markedly change the way water molecules vibrate, particularly in the librational and O-H stretch modes. We anticipate that advanced techniques such as correlated vibrational spectroscopy, a third-order nonlinear method based on hyper-Raman scattering (*73*), could yield crucial insights into interfacial hydrogen bonding and the vibrational dynamics of confined water between MXene layers.

### Water transport and correlation factors

**Fig. 5.**
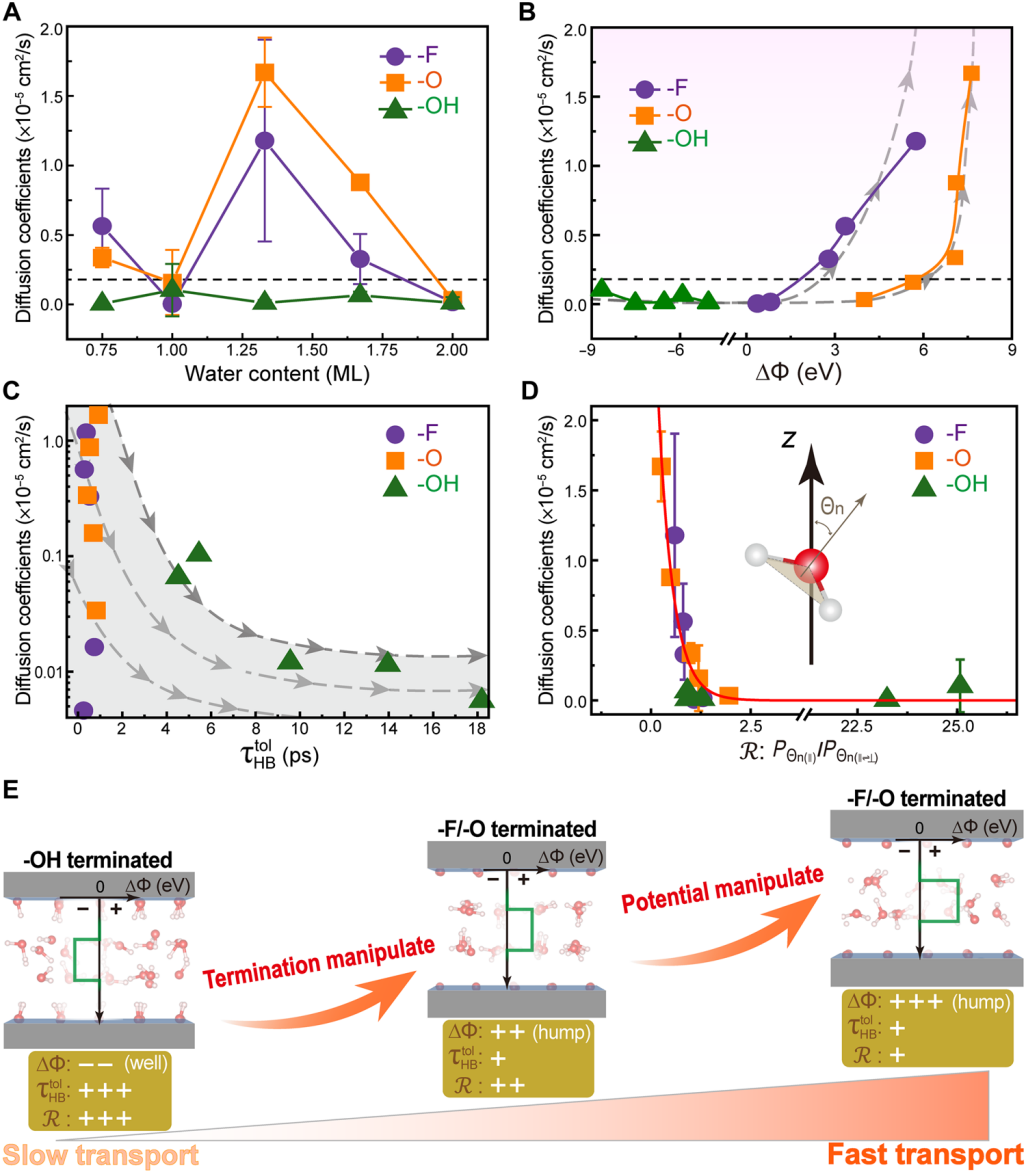
Water transport through MXene nanochannels. (**A**) Diffusion coefficients of confined water at varying water content. (**B**) Diffusion coefficients as a function of the electrostatic potential difference ∆Φ. The horizontal dashed line corresponds to the diffusion coefficient of the bulk water. (**C**) Diffusion coefficients versus total HBLTs $\tau_{\text{HB}}^{\text{tol}}$ for different surface groups. (**D**) Schematic of the $\theta_{n}$ angle between the *z* direction and the normal to the H-O-H plane and relationship between diffusion coefficients and the ratio R of $P_{\mathrm{\theta n}\left(∥\right)}$ to $P_{\mathrm{\theta n}\left(∥⇌⊥\right)}$. $P_{\mathrm{\theta n}\left(∥\right)}$ and $P_{\mathrm{\theta n}\left(∥⇌⊥\right)}$ represent the proportion of water molecules with a normal angle of 0° to 30° and 150° to 180° and 30° to 60° and 120° to 150°, respectively. (**E**) Schematic of how the identified key factors alter the water transport in MXene channels. The + and − signs of ∆Φ indicate the interfacial potential hump and well, respectively.

Theoretically, the diffusion coefficients (*D*) can be obtained from the mean-square displacement (fig. S18), allowing us to assess the diffusion behavior of water, as one of the most fundamental properties. As a reference, *D* of bulk water is calculated to ~0.18 × 10^−5^ cm^2^/s, agreeing well with the value from reported AIMD calculations (*74*). In Fig. 5A, the -F and -O terminated MXenes exhibit similar diffusion patterns with a single-peak feature during water intercalation, whereas the -OH terminated MXene shows suppressed diffusion, with *D* remaining below that of bulk water. Then, on the basis of these *D* values, the confined water can be classified to (i) fast diffusion in the -F and -O terminated MXenes and (ii) nearly unchanged but slow diffusion in the -OH terminated MXenes. The nonstoichiometric fractional water layers (such as 0.75, 1.33, and 1.67 ML) show faster diffusivity than 1 or 2 ML water. The fastest diffusion is observed at 1.33 ML water within the -O terminated MXene layers. It is thus exciting to observe that using either -F or -O surface groups, one could manipulate the water diffusion from almost frozen (-OH) to the ultrafast transport (-F/-O with fractional water layers). While in the earth’s deep interior, hydroxyl groups as the accommodator of the water could lead nominally anhydrous mineral to form a superionic channel for the H diffusion (*75*). We would like to recall that in the above analysis about the interlayer spacing, intercalation energy and charge density profiles at the SLI all point to a similar pattern for the -O and -F terminated MXenes. Similarly, the coincidence is also reproduced in the water diffusion, as shown in Fig. 5A. In the cases of 1.33 ML with either -O or -F terminations, the water shows a central coalesced region (Fig. 2C), illustrating that the water at the SLI is becoming more friction free.

The impact of the surface groups on the diffusivity could be derived via the inelastic/quasi-elastic neutron scattering. In fig. S19 (A and B), INS studies reveal a two–order-of-magnitude reduction of water self-diffusivity in the case of MXene sample having higher amount of -OH groups. As shown in fig. S19C, the pH-dependent zeta potential reveals systematic variations in both the magnitude and sign of the electrical potential during the termination transformation from -OH to -O terminations, well aligned with the increase and change in sign of ∆Φ presented in Fig. 3 (B and C). Both the INS and zeta potential results align with the theoretical trends, where large diffusion coefficients for -F/-O termination lead to faster diffusion than the -OH termination–driven diffusivity at the hydration levels with a noninteger number of water layers. This agreement establishes a foundation for potential future quantitative comparison and predictions, despite the existing theoretical limitations regarding the surface terminations.

In reality, thermal or some of sophisticated treatments (*32*, *76*–*78*) can effectively remove -OH groups to enhance water diffusion. The approximately theoretical estimates of the surface group stability were also performed. Figure S20 shows the calculated formation energy ${\Delta H}_{f}$ of the three surface groups (-F, -O, and -OH) in Ti_3_C_2_T_2_ as a function of the change in hydrogen chemical potential $\Delta \mu_{H}$. All formation energies exhibit large negative values, signifying energetically favorable bonding between the transition metals and all three surface groups. In principle, the enhancement of the pH value leads to a larger ratio of -O:-OH, as prediction that the -O surface groups exhibit the lowest ${\Delta H}_{f}$ for $\Delta \mu_{H}$ < −0.25 eV. Via the computational hydrogen electrode approach, we are able to plot surface Pourbaix diagrams as a function of pH and the applied electrode potential, as illustrated in fig. S21A. It indicates that the Ti_3_C_2_ surface with -O termination is stable above the hydrogen evolution reaction (HER) equilibrium line. Below the HER equilibrium, the -OH termination becomes favorable due to the proton reduction, while the -F termination is stable only within a rather narrow pH range near the HER line, specifically under strongly acidic conditions (pH ≤ 2). Conversely, under very negative potentials, the -H termination becomes more stable than the other terminations. It is worth noting that the bare surface exhibits poor stability. Our results are broadly consistent with the reported data in fig. S21B (*79*).

Considering that the confined water diffusion can be tuned by the electric field (*80*) and the reorientation of dipoles within the confined water (*81*), we pick up the SLI-related electrical and morphological features to gain a profound understanding of the water diffusion. As enlightened in Fig. 3 (B and C), the surface group would modify the interfacial charge and lead toward the negative ∆Φ in the -OH terminated cases contrasts with the positive and high magnitude of ∆Φ in the -O or -F dominant MXene surface. This charge modulation correlates with diffusion trends as shown in Fig. 5B, where *D* increases quadratically with ∆Φ at -F and -O terminations, and by contrast, *D* is almost invariant regardless of ∆Φ fluctuations at -OH terminated interfaces. In other words, when the confined water attains a higher electrostatic potential relative to the surface groups, a potential hump forms at the interface, enhancing the electrical activity of water and thereby promoting diffusion. A similar phenomenon is also observed in the confined water between α-Al_2_O_3_ sheets, where the enhanced in-plane diffusion is accompanied by the high surface potential and interfacial electric fields (*82*).

As presented in fig. S8, a higher number of the -OH groups make the surface more hydrophilic than the -O and -F groups, resulting in a more robust interaction with water. The additional HBs formed between -OH terminations and water (i.e., ${\text{HBs}}^{T-W}$) could thus influence the water diffusion as well (fig. S11D). The relationship between *D* and the total HBLT $\tau_{\text{HB}}^{\text{tol}}$ as shown in Fig. 5C qualitatively confirms a negative correlation between the HBLT with the water diffusion. The -OH that regulated a strong and long-lasting HB network freezes the water molecules, giving an ultralow *D*. In strong contrast, the diffusion in the -F and -O terminated cases can be ignited by localized stimulation due to the shorter HBLTs, and analogous alterations in the HBLTs and numbers culminate in a comparable diffusion response of water to ∆Φ, shown in Fig. 5B.

The interfacial potential difference and hydrogen bonding as above both fundamentally originate from the SLI and its effect on the morphology of water. The normal angle $\theta_{n}$ (Fig. 5D, inset) has been leveraged to quantify water structural ordering and orientation in the preceding section. Here, $\theta_{n}$ is further categorized and statistically analyzed in fig. S22, to assess the water morphology. When $\theta_{n}$ falls into the range of 60° to 120°, the water molecules are prone to orient perpendicular to the MXene surface (⊥), and between the range of 0° to 30° and 150° to 180°, the water molecules prefer to align parallel to the surface (∥). One last category (θ_n_ = 30° to 60° and 120° to 150°) defines the swing water, being the intermediate states of the above two (∥⇌⊥). Figure 5D reveals a notable exponential correlation between *D* and the ratio R of parallel to intermediate states. Maintaining more and more swing water molecules in the intermediate state can effectively enhance the diffusion of confined water within Ti_3_C_2_T_2_ MXene nanochannels, thereby improving mass transport.

Hereafter, through a meticulous and methodical analysis, we build up a multidimensional model to approximately describe the water diffusion as illustrated in Fig. 5E. The water layer exhibiting the highest *D* at each termination undergoes the most marked fluctuation in charge density, consistent with the physical picture of ∆Φ. In addition, the slow diffusivity in the -OH case could stem from the trapped water by the interfacial potential wells and the robust HB network at the interface. The orientational flip revealed in R is also related to the polarization fluctuation, and such water/polarization flip and the resulting friction between water and MXene could alter ∆Φ, which forms an interdependence relationship and correlation. With the above, we propose a model represented as $D=\alpha e^{a∆\Phi }+\beta e^{b\tau }+\gamma e^{\mathrm{cR}}$, where α, β, and γ denote weighting factors for the primary variables, and parameters *a*, *b*, and *c* are used to define each function of ∆Φ, $\tau_{\text{HB}}^{\text{tol}}$, and R. These findings emphasize the sophisticated interplay between the interfacial electrostatic potential, the water morphology (HBs and R), and the diffusivity of water.

While the main focus of this work has been on the conventional -F, -O, and -OH terminated Ti_3_C_2_T_2_ systems, recent advances in molten salt synthesis have enabled the synthesis of halogen-terminated MXenes, such as -Cl, which are experimentally accessible and exhibit distinct surface chemistry, interfacial properties, and water diffusions. Our extended analysis on the -Cl terminated MXene at 1.33 ML water content confirms that -Cl terminations can yield faster water diffusivity, driven by an enhanced interfacial electrostatic potential and weakened hydrogen bonding (fig. S23), thereby supporting the mechanistic framework established herein. Furthermore, considering that experimentally synthesized Ti_3_C_2_T*_x_* MXenes are often with mixed surface terminations, we also investigate representative mixed systems, specifically Ti_3_C_2_O_1.5_(OH)_0.5_ and Ti_3_C_2_O_1.42_Cl_0.58_, with termination ratios consistent with experimental reports (*22*, *32*). Despite incorporating about ~29% -Cl or ~25% -OH, confined water in these systems still exhibits higher diffusivity than bulk water, as illustrated in fig. S24. The reduced diffusivities compared to pure -Cl or -O systems are primarily due to a diminished interfacial potential difference, along with a slight decline in the orientational ratio R, while HBLTs remain comparably short (fig. S24). Together, these insights gained from the halogen and mixed surface terminations further validate our diffusion model.

In short, a more disordered or irregular framework tends to facilitate the commencement of water diffusion on a local level, as opposed to a structured or systematic setup where the diffusion starts more globally but with greater difficulty. Typically, the low potential for water, prolonged HBLTs, and a large R can considerably slow down the water diffusion and vice versa. Such interesting and insightful findings could inform prospects for purposefully directing water diffusion and conformational order via judicious selection of surface group species combined with external stimuli. Translating the correlation factors to the transport properties is highly possible and could forge an innovative approach to engineering the water behaviors. Recent work by Chen *et al.* (*83*) establishes a subcontinuum flow model with average density, viscosity, and slip length as the modified Navier-Stokes equation to explain the facilitated solvent permeation through sub–1-nm MXene-graphene heterostructure membranes. In parallel, the present work quantitatively correlates the SLI electrostatic potential, HBLT, and water orientation with the confined water transport between MXene layers, enriching the nanofluidic model with various media and scales. Complementarily, the experimentally observed gate-controlled water permeation in Ti_3_C_2_ MXene-based membranes under applied voltage further demonstrates the feasibility of controlling confined water diffusion via electrostatic potential (*84*). In general, we underscore the promising prospects tailoring a two-tiered control methodology, including surface groups and the manipulation of potential at SLI, to boost/freeze the water transport that are decisive to both electron double layer and/or redox dominant capacitances.

## DISCUSSION

To clarify the correspondence between simulations and experiments, the comparison in this study is interpreted at the trend level rather than as a strict one-to-one match. The pure termination models are used as bounding cases, because experimentally synthesized Ti_3_C_2_T*_x_* contains mixed terminations and inherent defects. As for the key structural response, namely, interlayer spacing, a semiquantitative agreement can be made. Two near-realistic Ti_3_C_2_O_1.5_(OH)_0.5_ and Ti_3_C_2_O_1.42_Cl_0.58_ MXenes show that both the structural and dynamical behaviors obtained from the bounding cases are rational, strongly relevant, and interpretable to experiments. The descriptor-diffusivity relationship captures the mechanistic trends, but the mapping between hydration, terminations, and descriptors shows that surface chemistry and hydration may in turn experimentally modify the related descriptors.

For practical electrochemical energy storage, the distribution and dynamics of intercalating cations, together with their hydration and interactions with the electrode surface, are key factors that determine capacitive performance (*35*, *85*). The present study of water intercalation serves as a baseline for investigating cation-containing electrolytes and understanding their hydration environments and dynamical behavior in MXene interlayers. Moreover, the diffusivity model developed here may be further refined for other aqueous electrolyte systems and ultimately evolve to a general description of water or hydrated ion transport in electrochemical devices.

Although developed for hydrophilic MXenes, the three descriptors in our diffusivity model are potentially applicable to other 2D confined water systems. Prior studies have shown that water confined between graphene slits exhibits fast diffusion accompanied by enhanced structural ordering (*16*, *86*), whereas in graphene oxide layers, diffusion becomes substantially slower due to electrostatic interactions and strengthened HB networks (*17*, *87*). External electric fields have also been shown to reorganize interfacial water structures (*81*) and modulate mobility in graphene oxide slits (*88*), CNTs (*89*), and folded silica sheets (*80*). Notably, the positive or negative correlation of each descriptor with the diffusivity depends strongly on surface chemistry. The ordering-induced slowdown observed in hydrophilic MXenes contrasts with the ordering-enhanced diffusion on hydrophobic graphene surfaces (*86*). Thus, while the present model may offer a transferable framework for confined water transport, the formula may require modification when applied beyond MXene systems.

In conclusion, we have systematically quantified the morphological changes along with the intercalation thermodynamic and kinetic behaviors of nanoconfined water between different functionalized MXenes, by means of MLaMD and advanced experimental characterizations. Our calculated structural changes associated with water intercalation agree well with the XRD measurements, showing a layer-dependent staging behavior. Beyond a water content of 1 ML, a bilayer distribution of water populations emerged, with water-water interactions predominantly governing the changes in the interlayer spacing. The effective densities of water in all cases are linearly correlated with the order parameter as well as the electrostatic potential changes at SLIs. Such unique distribution, morphology, and polarization of water lead to the electrostatic potential hump (-F, -O) and well (-OH) within the water layer across the MXene-water interfaces. The reduced HBLTs, together with the enhanced electrostatic potential and swing water molecules, considerably promote the water diffusivity in either -O or -F terminated cases. The termination-dependent diffusivity and electrostatic potential are both aligned with the neutron scattering and the zeta potential measurements. Overall, these insights lay the groundwork for regulating the water transport by tuning MXene surface groups and controlling water intercalation, enabling exponential model-based optimization for water transport and water-mediated cation or proton highways in superior charge storage devices. This work further opens avenues for designing high-power batteries/supercapacitors, membranes with programmable selectivity, and next-generation desalination channels with atomic-precision control.

## MATERIALS AND METHODS

### Computational methods

All DFT calculations and MLaMD simulations were conducted using the Vienna Ab initio Simulation Package (VASP) software package (*90*). The generalized gradient approximation in the form of the Perdew-Burke-Ernzerhof functional was used to address electron exchange-correlation, in conjunction with the dispersion correction method DFT-D3 to account for van der Waals interactions (*91*, *92*). The ion-electron interactions were described using the Blöchl projector augmented wave method within the frozen-core approximation, along with the plane-wave basis sets (*93*, *94*). To model the MXene-water interfaces, different contents of water ML were inserted into Ti_3_C_2_T_2_ (T = -F, -O, and -OH) supercells containing 12 formula units (0.25, 0.5, 0.75, 1, 1.33, 1.67, and 2 ML corresponding to 3, 6, 9, 12, 16, 20, and 24 water molecules, respectively). After a careful convergence test on the orthorhombic supercell, a high energy cutoff of 500 eV and a 3 × 3 × 2 Γ-centered Monkhorst-Pack k-mesh were found to be sufficient for converging the total energy within 1 meV per atom. For structural optimizations, the self-consistence convergence criterion was set to 10^−5^ eV for energy, and the ionic convergence criterion was set to 0.02 eV/Å per ion for the Hellman-Feymann force. Specifically, the interlayer spacing and atomic positions of interface structures were fully relaxed until thermodynamic equilibrium, serving as input structures for subsequent dynamic simulations. To validate the results, we further plotted the average surface force as a function of interlayer spacing (fig. S1). In all cases, the forces converge to their respective minima near zero at the optimized spacing, confirming the reliability of the calculated values.

High-accuracy AIMD simulations are usually constrained to small simulation cells and durations. The recently developed on-the-fly MLaMD method offers a unique solution, expediting calculations by autogenerating MLFF through Bayesian inference in MD simulations, with first-principles calculations executed only when new configurations emerge from previously sampled datasets (*95*, *96*). This approach can speed up the AIMD simulations by over two orders of magnitude while ensuring near AIMD-level accuracy in the simulation outcomes (*97*, *98*). During MLaMD simulations, the NVT ensemble (constant number of atoms, constant volume, and constant temperature) combined with Nosé-Hoover thermostat for temperature control (*99*, *100*) were used, in a time step of 1.5 fs. At 300 K, an initial 15 ps was used for system equilibration, ensuring proper balance, while the additional 30 ps was dedicated to sampling and analysis of the interfacial system. The convergence behaviors of the temperature and energy during MLaMD simulations are shown in fig. S2. To evaluate the averaged electrostatic potential and charge density distributions, equilibrium trajectories were collected every 0.75 ps from the last 15 ps of the 45-ps simulation, yielding a total of 21 frames. For each of these frames, self-consistent DFT calculations were performed to obtain the electrostatic potential and charge density distributions, and the presented results can be obtained by averaging over all frames.

### Experimental details

Ti_3_AlC_2_ powder (1 g) was gradually added to a solution of LiF (1.6 g) and 9 M HCl (20 ml), and the mixture was stirred at 35°C for 24 hours. The etched material was then repeatedly washed with DI water through centrifugation until the supernatant reached a pH of 6. The sediment was dispersed in 40 ml of DI water and sonicated for 1 hour under argon. After centrifugation at 3500 rpm for 1 hour, the colloidal solution of few-layered Ti_3_C_2_T*_x_* was collected, filtered into flexible films, and dried under vacuum at room temperature for subsequent treatment and analysis.

XRD using Cu-Kα radiation (λ = 1.54 Å) was performed to assess the interlayer structure of pristine and charged Ti_3_C_2_T*_x_* films with various hydration states. In situ XRD was performed as the temperature increased from room temperature to 450°C. XPS was performed using a Thermo Fisher Scientific K-Alpha spectrometer with a monochromatic Al Kα (1486.6 eV) source at 36 W, with all peaks corrected for charge shift using the C1s hydrocarbon peak at 284.8 eV. The INS spectra of MXene samples were obtained using the Fine Resolution Fermi Chopper Spectrometer (*101*, *102*) with incident neutron energies (*E*_i_) of 250 meV at the Spallation Neutron Source, Oak Ridge National Laboratory. More experimental details can be found in the Supplementary Materials.

## Supplementary Materials

### This PDF file includes:

Supplementary Text S1 to S13 Figs. S1 to S24 Tables S1 and S2 References
