# Supplementary material for: Altered morphology and diffusivity of water confined in MXenes: Machine learning–accelerated computations combined with experiments
Source: Sci Adv. 2026 Mar 25;12(13):eadz1780. doi: 10.1126/sciadv.adz1780 (PMC13015896; doi:10.1126/sciadv.adz1780)
Supplement: Supplementary file 1 — Supplementary Text S1 to S13 Figs. S1 to S24 Tables S1 and S2 References [file sciadv.adz1780_sm.pdf]

Science Advances
AAAS

Supplementary Materials for

Altered morphology and diffusivity of water confined in MXenes: Machine learning–accelerated computations combined with experiments

Jiawei Tang *et al.*

Corresponding author: Weiwei Sun, provels8467@gmail.com; Xuehang Wang, x.wang-22@tudelft.nl

Sci. Adv. **12**, eadz1780 (2026)
DOI: 10.1126/sciadv.adz1780

This PDF file includes:

Supplementary Text S1 to S13
Figs. S1 to S24
Tables S1 and S2
References

Supplemental Text 1. Thermodynamic and dynamic equilibrium of the interfaces

To validate the accuracy of the presented interlayer spacing, we compute the surface force as a function of distance to identify the minima of the interlayer spacing, as shown in Fig. S1. The determined interlayer spacing values align well with the results by the full optimization, as shown in Fig. 1C.

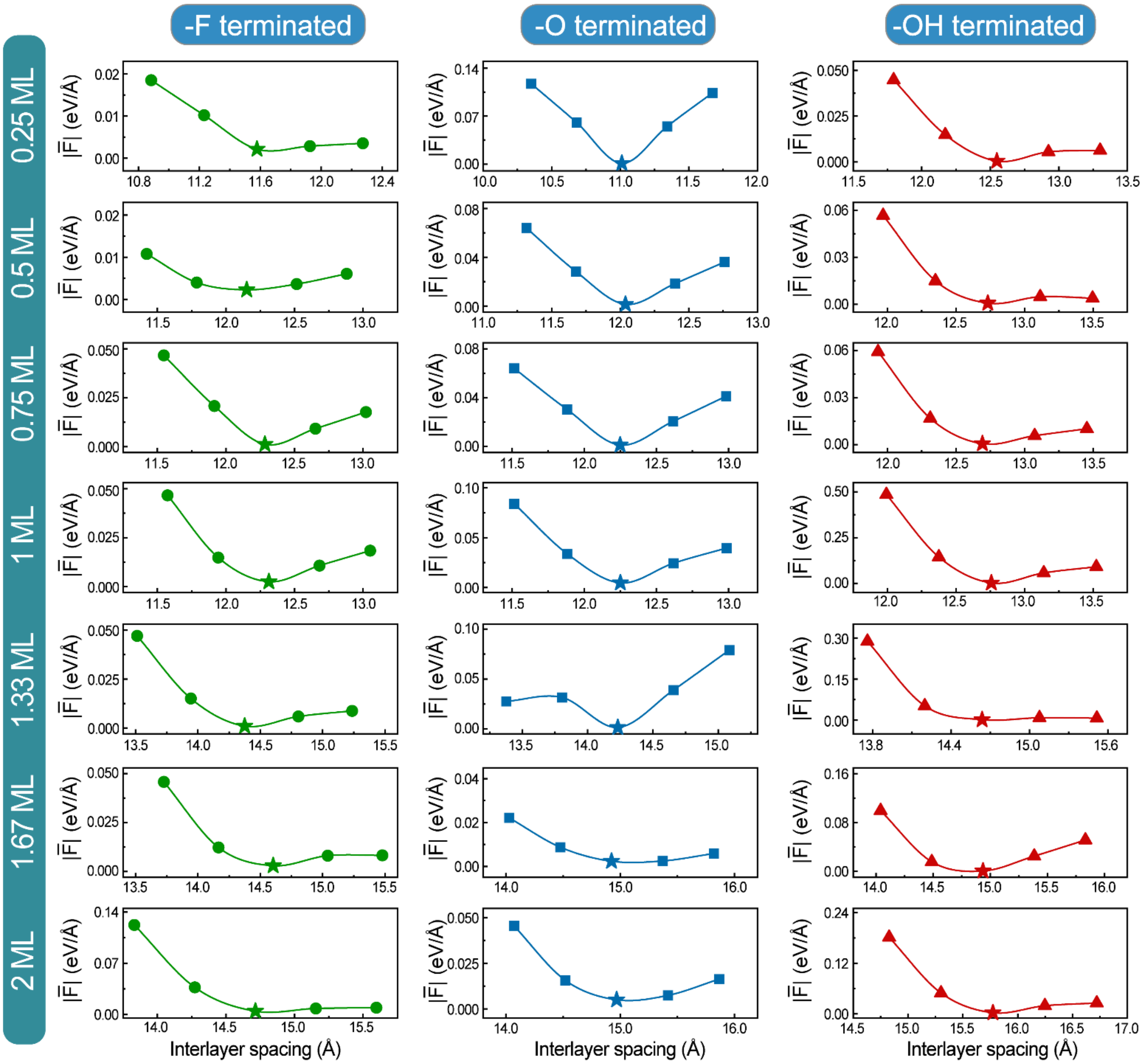

Fig. S1. Verification of model reliability. Average force on surface group atoms as a function of interlayer spacing. The stars represent the interlayer spacing values used in this work.

To ensure reaching upon the corresponding equilibrium states of water intercalated MXenes, we extended the machine learning-accelerated molecular dynamics (MLaMD) to 45 ps. And the temperature (T) and energy (E) progression over the last 30 ps at 300 K is shown in Fig. S2.

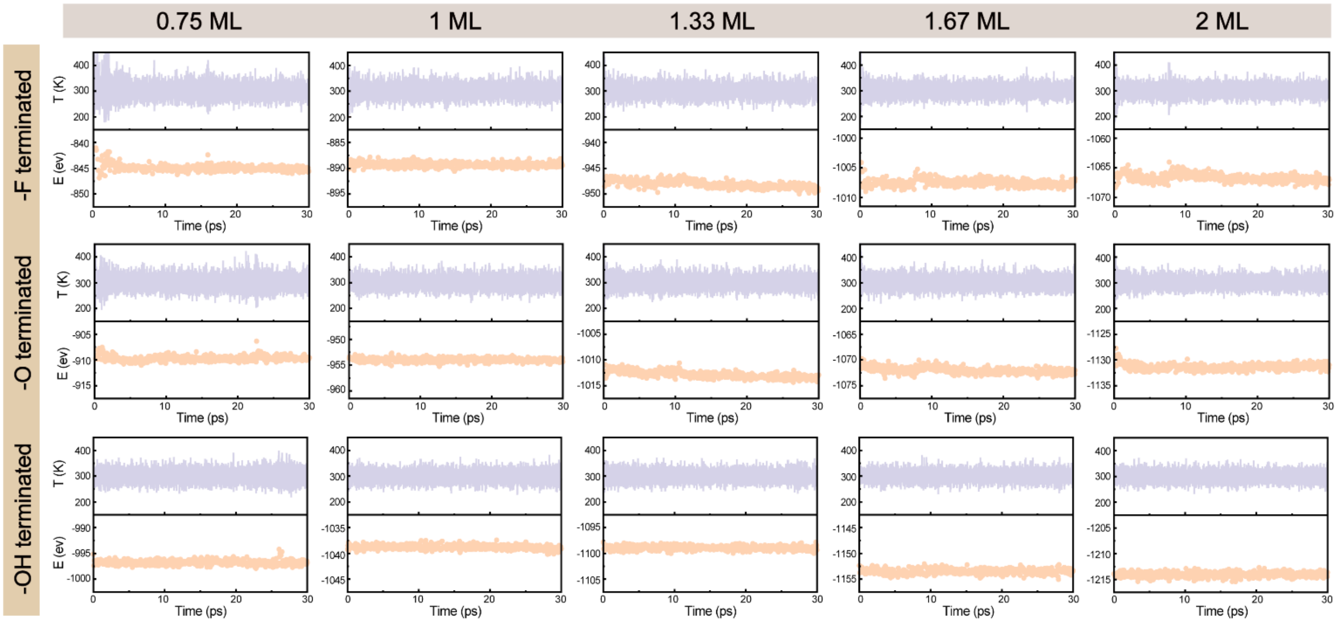

Fig. S2. Temperature and energy curves during MLaMD. The evolution of temperature (T) and energy (E) during the 30 ps MLaMD at 300 K for MXenes with varying water content.

Supplemental Text 2. Water intercalation

2.1 Energetic decomposition analysis at -OH terminated interfaces

The intercalation energy ΔE_{int} can be decomposed into three components as shown in Fig. S3A: (1) $\Delta E_{(MXene)}$, the energy cost of deforming the MXene structure, (2) $\Delta E_{(Water)}$, the energy associated with structuring water molecules into a confined layer, and (3) $\Delta E_{(MXene-Water)}$, the energy gain from interfacial coupling. This decomposition allows us to dissect the increase in ΔE_{int} observed at 1 and 2 ML for -OH terminated MXenes. As summarized in Table S1, when the water content increases from 0.75 ML to 1 ML, $\Delta E_{(MXene-Water)}$ becomes more negative, indicating the occurrence of a strong interfacial coupling. However, both $\Delta E_{(MXene)}$ and $\Delta E_{(Water)}$ increase markedly compared with their values at 0.75 ML, offsetting the interfacial energy gain and leading to the anomalous rise in ΔE_{int}. Structural analyses further reveal the microscopic origins of this increase. As shown in Fig. S3B, the pair distribution function (PDF) of terminal (O_T) and water oxygen (O_W) $g_{O_T-O_W}$ shifts to shorter distances at 1 ML, consistent with enhanced interfacial coupling rather than steric exclusion by -OH groups. For confined water, the disappeared peak at 4~5 Å in $g_{O_W-O_W}$ reflects a less compact water structure, resulting in the increased $\Delta E_{(Water)}$. Meanwhile, the g_{O_T-H} displays a clear shift in the range of 2~2.8 Å, confirming the reorientation of -OH groups at 1 ML, and the broadening of the g_{Ti-O_T} peak near 2 Å reflects the

elongation of Ti-O bonds. These surface distortions and water reorganizations collectively account for the increase in ΔE_{int} at 1 ML.

Regarding the 2 ML water, $\Delta E_{(MXene)}$ and $\Delta E_{(Water)}$ both decrease relative to their values at 1.67 ML, $\Delta E_{(MXene-Water)}$ becomes significantly less negative. The reduced energy gain from interfacial coupling thus accounts for the observed increase in ΔE_{int} at this case. This interpretation is corroborated by pair distribution functions (Fig. S3C), which reveal enlarged O_W-O_W distances and a more ordered water structure at 2 ML, both indicative of weakened MXene-water interactions.

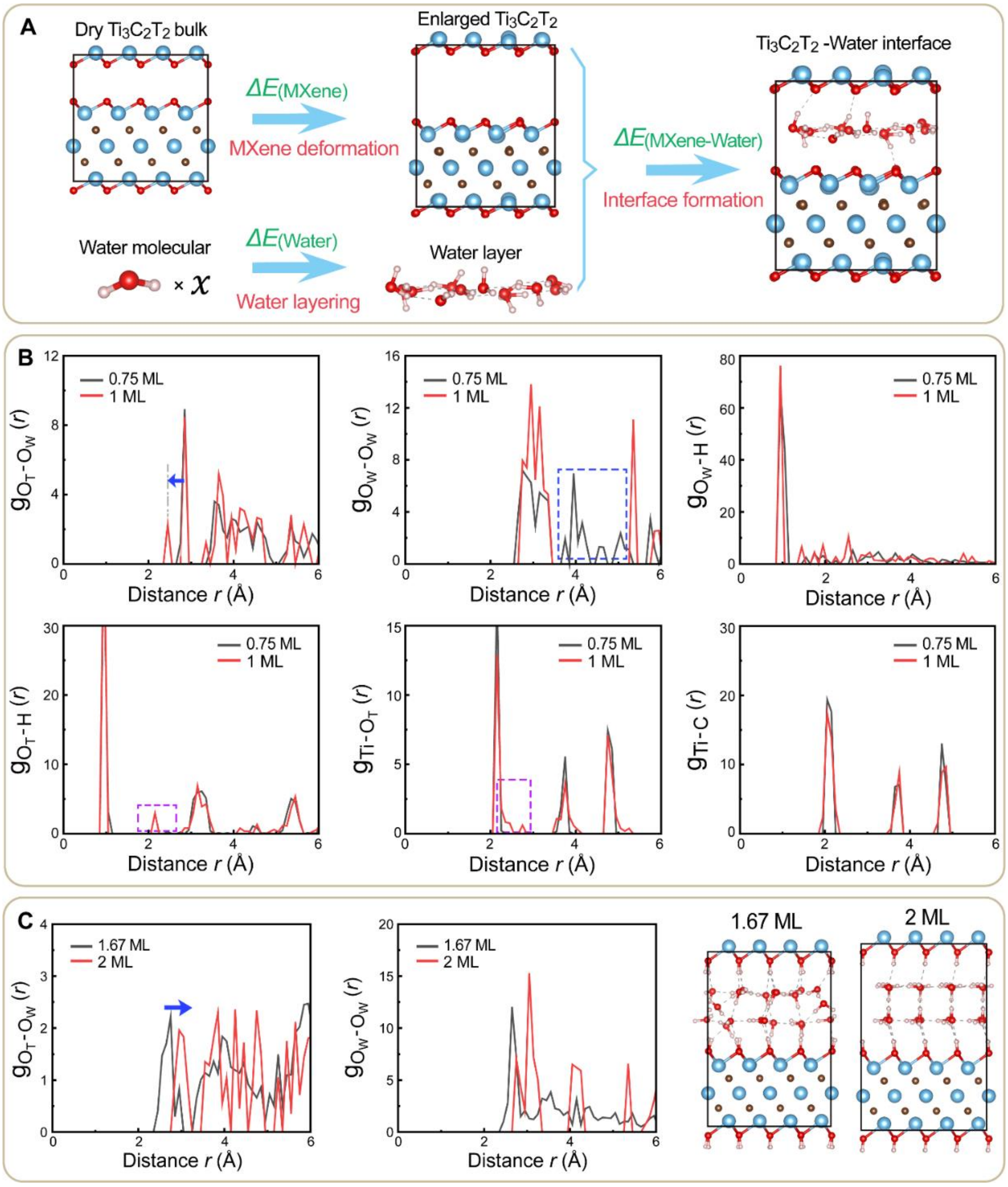

Fig. S3. Decomposition analysis of intercalation energy. (**A**) Schematic decomposition of intercalation energy ΔE_{int}, including contributions from MXene structural deformation, water layering, and interfacial formation. (**B**) Pair distribution functions (PDFs) of $Ti_3C_2(OH)_2$ MXene at 0.75 ML and 1 ML water loading. (**C**) PDFs and typical snapshots of $Ti_3C_2(OH)_2$ MXene at 1.67 ML and 1 ML water. O_T denotes terminal oxygen atoms of MXene, and O_W denotes oxygen atoms of water molecules.

Table S1. Calculated $\Delta E_{(MXene)}$, $\Delta E_{(Water)}$, and $\Delta E_{(MXene-Water)}$, together with the resulting intercalation energy ΔE_{int}, for -OH terminated MXenes. (Unit: eV/H_2O)

Water content	$\Delta E_{(MXene)}$	$\Delta E_{(Water)}$	$\Delta E_{(MXene-Water)}$	ΔE_{int}
0.75 ML	0.16	-0.10	-0.85	-0.80
1 ML	0.39	0.36	-1.45	-0.70
1.33 ML	0.22	0.41	-1.62	-0.99
1.67 ML	0.43	0.55	-2.01	-1.03
2 ML	0.09	-0.51	-0.20	-0.62

2.2 Entropy for confined water

To assess the role of entropy under confinement, we employed the DoSPT program (*103*) based on the two-phase thermodynamic (2PT) model proposed by Goddard *et al.* (*104-106*) to calculate the relative entropy (ΔS) of confined water with respect to bulk, where $\Delta S = S_{confined} - S_{bulk}$. Both $S_{confined}$ and S_{bulk} consist of translational, rotational, and vibrational contributions. The calculated entropy of bulk water (S_{bulk}) is 62.9 $J \cdot mol^{-1} \cdot K^{-1}$, consistent with the experimental value of 69.9 $J \cdot mol^{-1} \cdot K^{-1}$. As shown in Fig. S4A, the relative entropy ΔS of confined water remains below zero, indicating reduced molecular freedom under confinement, and exhibits no systematic dependence on either surface termination or water content. The magnitude of $T\Delta S$ (Fig. S4, B to D) is roughly one order of magnitude smaller than the corresponding ΔE_{int} across all surface terminations. Therefore, the incorporation of entropy would only cause slight shifts in the ΔE_{int} curves, without affecting the overall trends or conclusions.

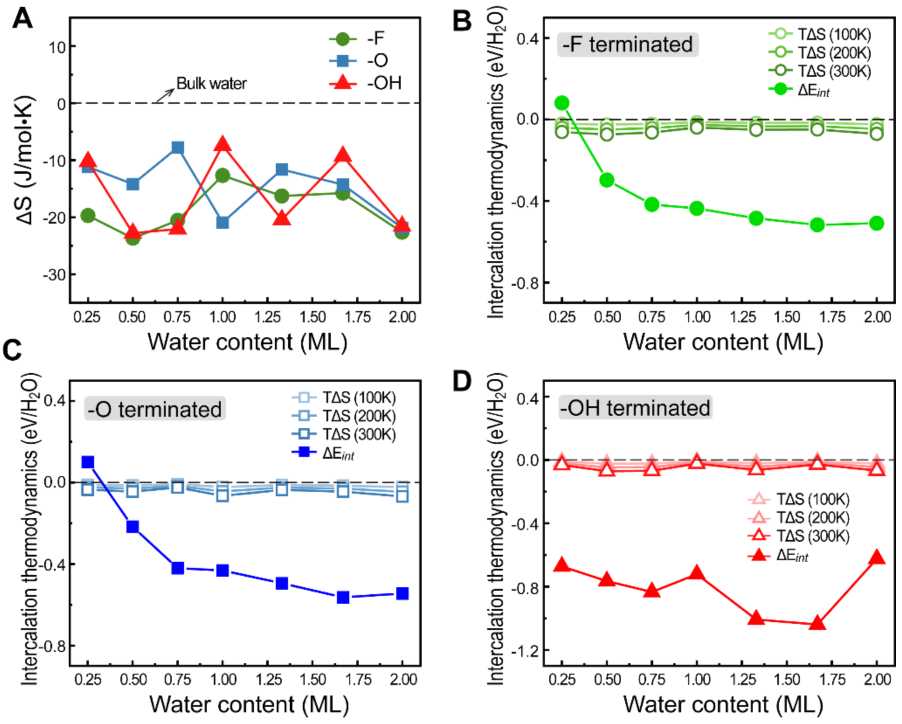

Fig. S4. Analysis of intercalation thermodynamics. (**A**) Relative entropy ΔS ($\Delta S = S_{confined} - S_{bulk}$) of confined

water as a function of water content. Comparison of the entropic ($T\Delta S$) and intercalation energy (ΔE_{int}) contributions for (**B**) -F, (**C**) -O, and (**D**) -OH terminated MXene, respectively.

Supplemental Text 3. Experimental synthesis and measurement

3.1 Synthesis of $Ti_3C_2T_x$ MXene

1 g of Ti_3AlC_2 was gradually introduced into a mixing etchant solution composed of 1.6 g LiF and 20 ml 9 M HCl. The mixture was stirred at 35°C for 24 hours in an oil bath. Subsequently, the etched product was repetitively washed with DI water through centrifugation until the pH of the supernatant reached 6. The resulting sediment was then dispersed in 40 ml DI water and subjected to sonication for 1 h under argon. Following this, the suspension underwent centrifugation at 3500 rpm for 1 h, and the colloidal solution of few-layered $Ti_3C_2T_x$ was collected, which was then filtrated into flexible films and dried under vacuum at room temperature (RT) for further analysis.

Direct modulation of surface chemistry can be achieved experimentally by tuning the -OH/-O ratio through electrochemical methods. Although high -OH coverage can be obtained via $NaBF_4$ hydrolysis (*107*), alkaline exchange of -F/-Cl (*108*), or atomic-hydrogen treatments (*109*), these routes often require harsh conditions or risk introducing structural modifications. For this reason, we employed electrochemical reduction as a cleaner and more controllable approach. In our study, -OH rich $Ti_3C_2T_x$ was prepared in 1 M H_2SO_4 by applying -0.8 V vs. Ag for 1 h, followed by water rinsing and vacuum drying at RT.

3.2 XPS measurement

X-ray photoelectron spectroscopy (XPS) was conducted using a Thermo Fisher K-Alpha surface spectrometer. The X-ray source employed was a monochromatic Al Kα (1486.6 eV) operated at 36 W (12 kV, 3 mA). The binding energy (BE) of C1s and O1s was further analyzed. All peaks are corrected for charge shift using the primary C1s hydrocarbon peak at a reference value of BE = 284.8 eV.

The charged $Ti_3C_2T_x$ sample was prepared by charging $Ti_3C_2T_x$ film at -0.8 V vs. Ag for 1 h in 1 M H_2SO_4, which was then washed with DI water to remove the electrolytes. The sample was dried at RT under vacuum for 12 h before XPS measurement. The XPS survey spectra (Fig. S5) demonstrate the

existence of Ti, C, O, F and Cl in both the pristine and hydroxyl-rich samples. The quantitative elemental analysis (Table S2) reveals only minimal -Cl groups in both samples. For the high-resolution spectra of C1s, the peaks can be fitted into four components: 282.1 eV (Ti-C), 283.98 eV (C-Ti-O), 284.8 eV (C-C), 286.34 eV (C-O) for pristine $Ti_3C_2T_x$ and 281.96 eV (Ti-C), 283.24 eV (C-Ti-O), 284.8 eV (C-C) and 286.49 eV (C-O) for the charged $Ti_3C_2T_x$. Similarly, the O1s spectrum can be fitted into four peaks: 529.75 eV (Ti-O), 531.51 eV (C-Ti-O), 532.65 eV (C-Ti-OH), 533.73 eV (C-O) for pristine $Ti_3C_2T_x$ and 529.73 eV (Ti-O), 530.88 eV (C-Ti-O), 532.15 eV (C-Ti-OH) and 533.47 eV (C-O) for the charged $Ti_3C_2T_x$. The ratio between hydroxyl groups and -O terminations on MXene surfaces can be estimated from the peak area of C-Ti-OH and C-Ti-O. A higher ratio of 1: 0.62 was obtained for charged $Ti_3C_2T_x$, compared to that of pristine $Ti_3C_2T_x$ (1:1), which confirmed the charged MXene is hydroxyl-rich, as more -O is transformed into -OH upon charging and H^+ intercalation.

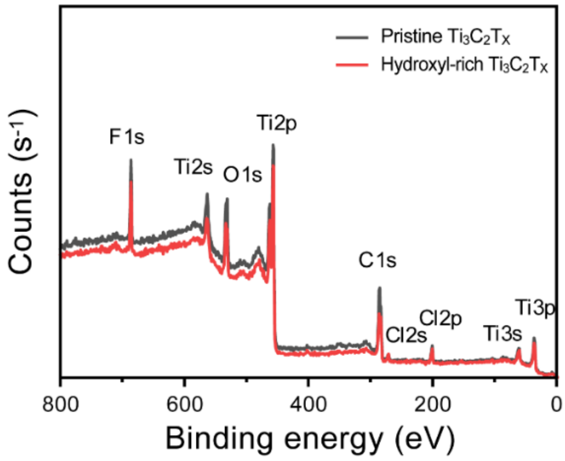

Fig. S5. XPS survey scan. XPS survey spectra of the pristine and hydroxyl-rich $Ti_3C_2T_x$ MXenes.

Table S2. Elemental composition from XPS survey spectra of pristine and hydroxyl-rich $Ti_3C_2T_x$ MXenes.

Sample	Ti (atom%)	C (atom%)	O (atom%)	F (atom%)	Cl (atom%)
Pristine $Ti_3C_2T_x$	24.9<br>(-1, +0.6)	46.0<br>(-0.7, +1.2)	16.2<br>(-0.2, +0.1)	9.8<br>(-0.1, +0.2)	3.1<br>(0.2, +0.1)
Hydroxyl-rich $Ti_3C_2T_x$	23.5<br>(-1.2, +0.9)	44.9<br>(-2.2, +2.1)	18.4<br>(-2.9, +1.7)	8.8<br>(-0.7, +0.8)	4.4<br>(-1.4, +0.9)

3.3 XRD measurement

X-ray diffraction (XRD) was employed to analyze the interlayer structure of pristine $Ti_3C_2T_x$ and charged Ti_3C_2 film utilizing an X’Pert Pro diffractometer from PANalytical, operating at 45 kV and 40 mA, with Cu-Kα (λ = 1.54 Å) radiation. In addition to freshly prepared samples, films with different

hydration states were also examined, including samples dried at 80 °C or 200 °C under vacuum for 12 h and those stored in a desiccator for nine months.

We further performed in-situ XRD of $Ti_3C_2T_x$ film under vacuum across a temperature range from RT to 450°C to examine changes in the interlayer spacing of MXene during water/OH removal (as shown in Fig. S6). As the temperature increased from 25°C to 150°C, a discernible rightward shift and broadening of the (002) diffraction peak were observed, indicating a gradual decrease in interlayer spacing and greater disorder. This behavior also aligns with the dynamic evolution of the (004) diffraction peak. The process corresponds to the loss of physically adsorbed water (*51*). This agrees with the simulation observation that the removal of water leads to interlayer spacing shrinkage. Further elevating heating beyond 200°C resulted in the loss of -OH groups from MXene, as evidenced by the emergence of a new peak at $2\theta = 7.8^{\circ}$, which remained unchanged up to 450°C. This new peak corresponds to a decreased *d*-spacing of MXene to 11.3 Å, consistent with simulations showing that fewer -OH groups lead to reduced interlayer spacing.

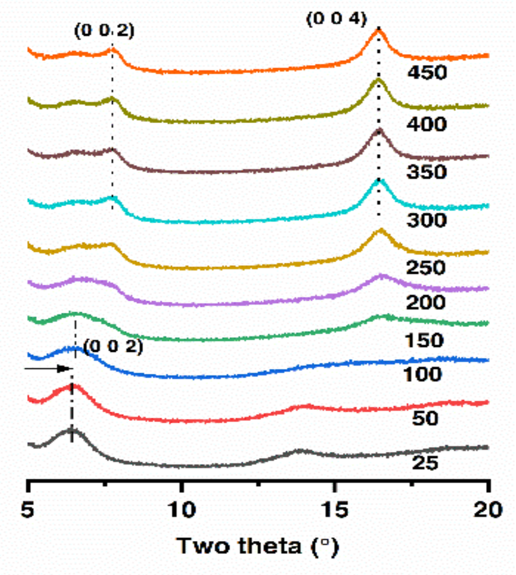

Fig. S6. In-situ XRD analysis. In-situ XRD of $Ti_3C_2T_x$ MXene heated at different temperatures.

Supplemental Text 4. Thermal effects on water de-intercalation

As the reverse process of water intercalation, the energy cost for the de-intercalation of each water molecule, called the de-intercalation energy, is defined as follows:

$$\Delta E_{de-int} = \frac{E_{tot}(Ti_3C_2T_2) + x \cdot E_{tot}(H_2O) - E_{tot}(Ti_3C_2T_2 \cdot xH_2O)}{x}, \qquad \text{(S1)}$$

where $E_{tot}(Ti_3C_2T_2)$ is the energy of dry $Ti_3C_2T_2$ bulk, $x \cdot E_{tot}(H_2O)$ is the energy sum of x isolated water molecules, and $E_{tot}(Ti_3C_2T_2 \cdot xH_2O)$ is the total energy of the hydrated MXene system

incorporating *x* water molecules.

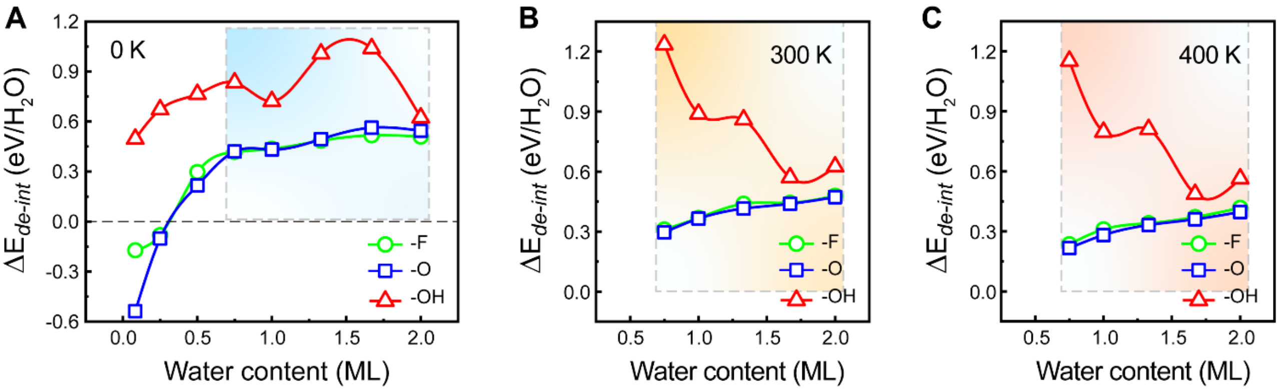

Fig. S7. Temperature-dependent de-intercalation energies. De-intercalation energy per water molecule as a function of water content at (**A**) 0 K, (**B**) 300 K, and (**C**) 400 K.

Supplemental Text 5. Hydrophilicity and interfacial hydrogen bonds

The calculated adsorption energies of H_2O on the three surface terminations are presented in Fig. S8A. These values follow the sequence $E_{ads}(-F) \geq E_{ads}(-O) > E_{ads}(-OH)$, indicating a progressively stronger water affinity, with -OH surfaces exhibiting the most negative adsorption energy and therefore the highest hydrophilicity. Consistently, the interfacial hydrogen-bond lengths (Fig. S8B) also show a termination-dependent trend aligned with the observed sequence of exclusion-volume thickness: $d_{-F} \geq d_{-O} > d_{-OH}$. The agreement among adsorption energy, hydrogen-bond length, and exclusion thickness collectively demonstrates that the enhanced hydrophilicity of the -OH termination is the fundamental factor underlying the reduced *d*.

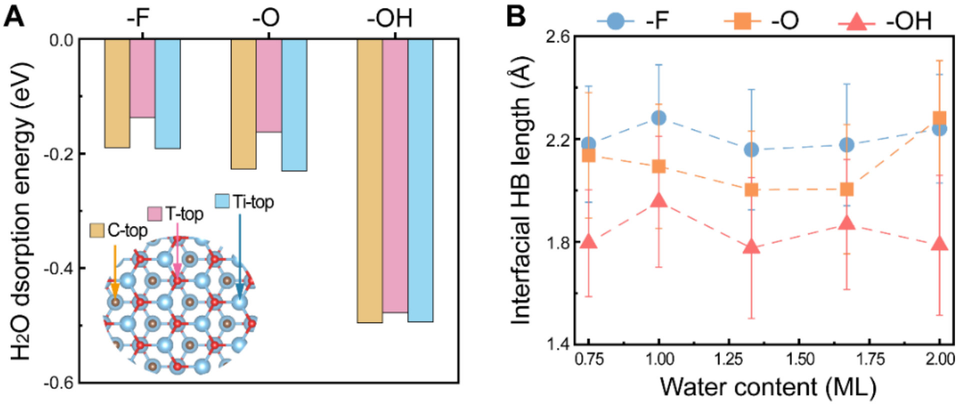

Fig. S8. Comparative analysis of hydrophilicity and interfacial hydrogen bonds. (**A**) Adsorption energy of H_2O on the three terminated MXene surfaces. The C-top, T-top, and Ti-top represent the adsorption sites located above the top sites of C atoms, terminated atoms, and Ti atoms, respectively. (**B**) Comparison of interfacial hydrogen bond lengths for -F, -O and -OH terminated systems.

Supplemental Text 6. Effective volume of confined water layers

The effective volume defined as the product of the effective height h_{eff} of confined water layer and the interfacial area A is shown in Fig. S9.

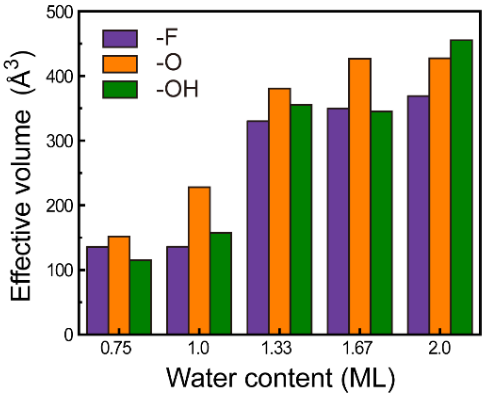

Fig. S9. Effective volume of confined water layers. Effective volume ($A \cdot h_{\mathrm{eff}}$) of confined water layers as a function of water content for different surface terminations.

Supplemental Text 7. Electrostatic potential

We further calculated the electrostatic potential across the MXene-water interface, which serves as another representative descriptor of the solid-liquid interface (SLI), as shown in Fig. S10. The variations in the potential profile, such as humps or dips between the two sides, offer valuable insights into the interfacial characteristics.

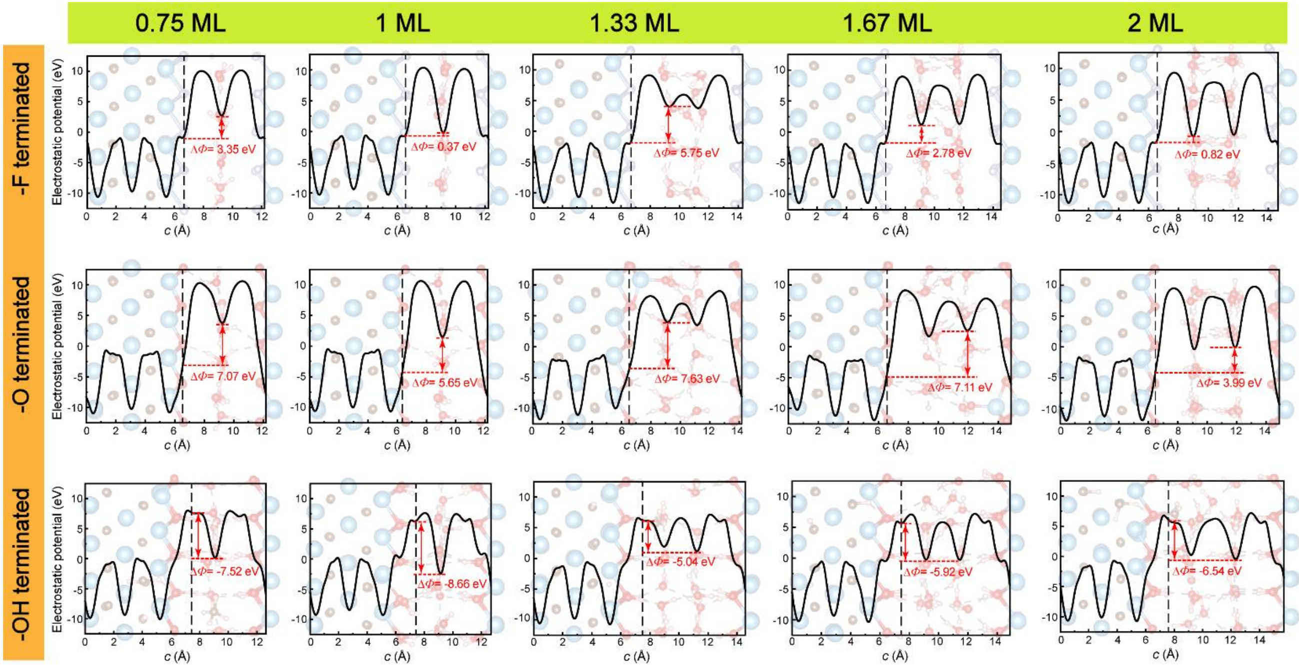

Fig. S10. Electrostatic potential distributions at MXene-water interfaces. The electrostatic potential profile of $Ti_3C_2F_2$, $Ti_3C_2O_2$, and $Ti_3C_2(OH)_2$ MXenes with varying water content.

Supplemental Text 8. Hydrogen bonds (HBs)

8.1 Average number of HBs per molecule

The hydrogen bonding between surface terminations and water is pivotal in distinguishing the intrinsic differences in MXene-water interactions for -OH and -F/-O terminated systems. Fig. S11A shows the prototypes of 2-HB, 3-HB and 4-HB from the equilibrated configurations. And the number of HB within the confined water, the HB contributed by the water to the MXene surface, as well as the HB exclusively presented in the -OH terminated cases are detailed in Fig. S11 (B to C).

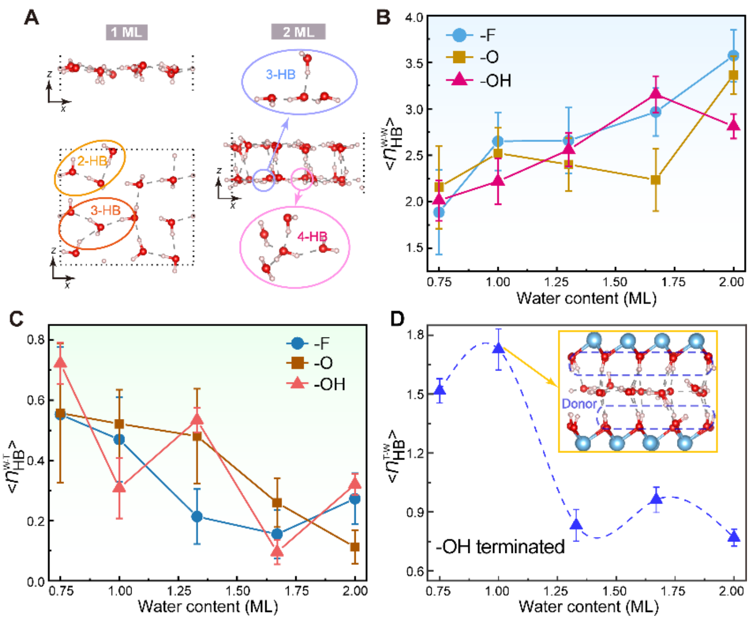

Fig. S11. HB structure and number analysis for each water molecule. (**A**) Snapshots of the HB configurations within 1 and 2 ML water. Average number of HBs per molecule, established (**B**) within confined water $\langle n_{HB}^{W-W} \rangle$, (**C**) between confined water (donor) and surface terminations (acceptor) $\langle n_{HB}^{W-T} \rangle$, and (**D**) between -OH terminations (donor) and confined water (acceptor) $\langle n_{HB}^{T-W} \rangle$.

8.2 HB interaction energy H_2O

To elucidate thermodynamics related to the HB interaction, we calculate the HB interaction energy as a function of water content, expressed below:

$$E_{HB} = \frac{E_{tot}(Ti_3C_2T_2 \cdot xH_2O) - E_{tot}(Ti_3C_2T_2) - x \cdot E_{tot}(H_2O)}{N_{HB}}, \quad \text{(S2)}$$

where $E_{tot}(Ti_3C_2T_2 \cdot xH_2O)$ is the total energy of the hydrated MXene system incorporating x water molecules, $E_{tot}(Ti_3C_2T_2)$ is the energy of $Ti_3C_2T_2$ free surface statically calculated using the same supercell size as above, $x \cdot E_{tot}(H_2O)$ is the total energy of x isolated water molecules, and N_{HB} is the total number of HBs.

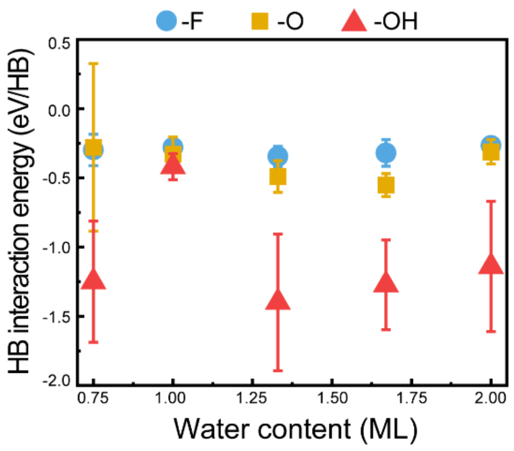

Fig. S12. HB thermodynamics. HB interaction energy per water molecule as a function of water content.

8.3 HB networks

Different types of SLIs and variances in intercalated water content are anticipated to result in different microstructures of water molecules. Here, we employ the dimensionality and the polyhedrality to characterize the water morphology, as shown in Fig. S13.

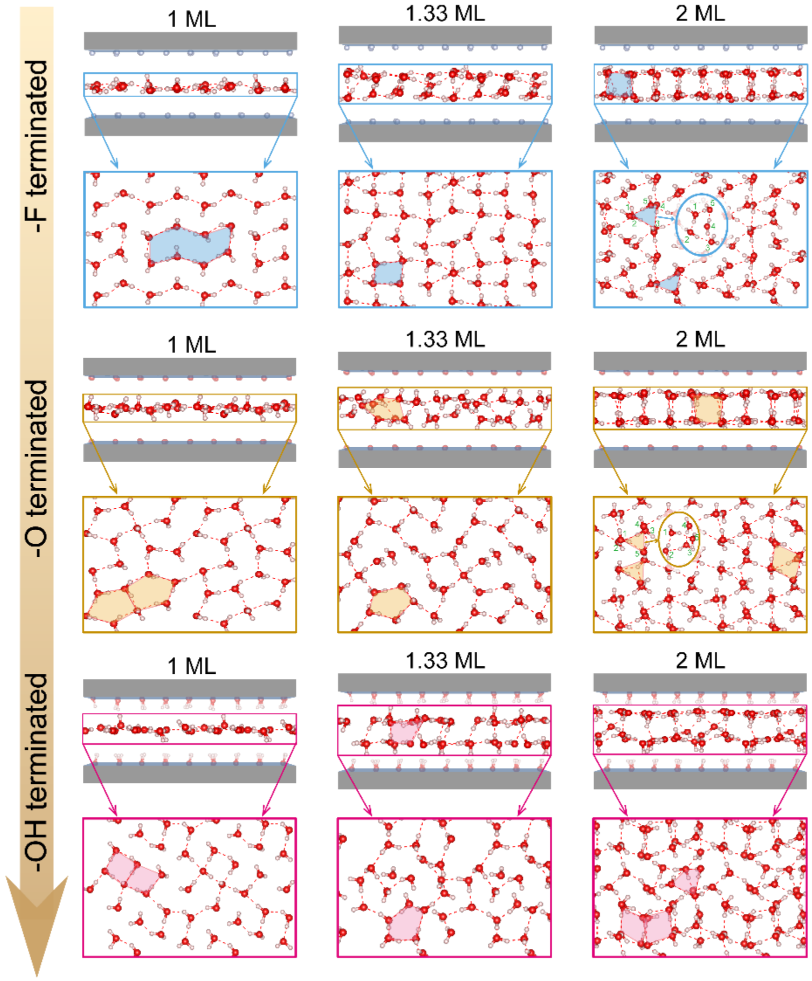

Fig. S13. HB networks within confined water. Comprehensive schematic diagram of the interlayer-confined water structure at varying water content.

In addition to the θ_n, the angle between the z-axis and the normal axis to the H-O-H plane of water molecules, we specifically analyzed the HB orientations at the interface and within the confined water layer, quantified by the angle θ_{HB}, defined as the angle between a single acceptor-H bond and the z-axis, in order to analyze the direction of HB, as shown in Fig. S14.

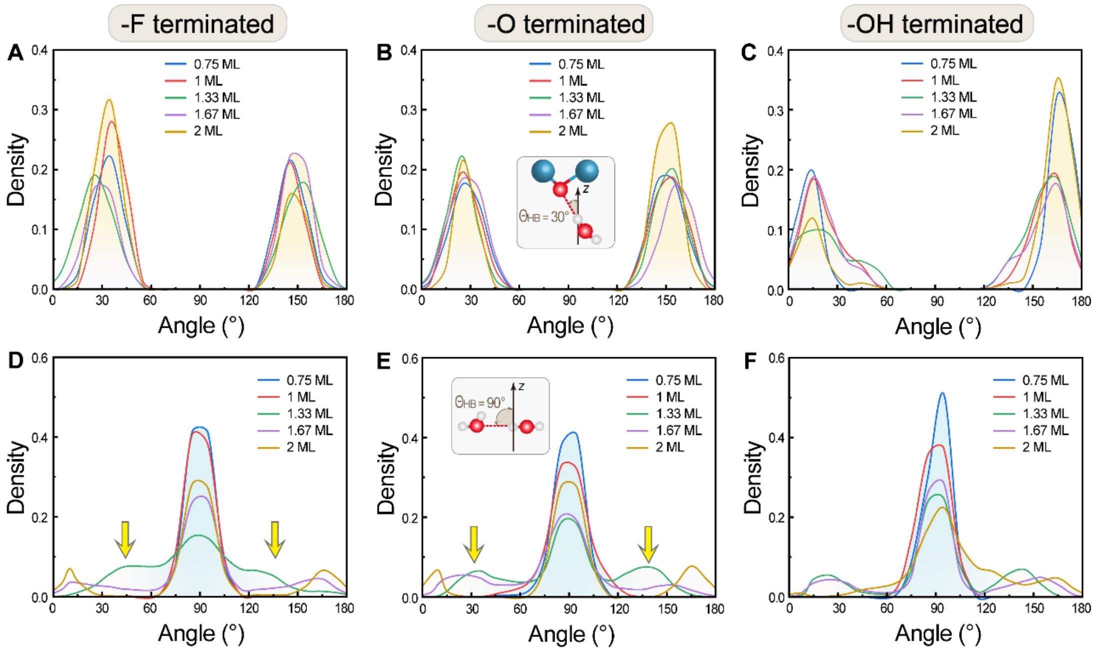

Fig. S14. The orientation analysis of HBs. θ_{HB} is defined as the angle between the acceptor-hydrogen bond and the z-axis. (**A**)-(**C**) show interfacial HBs for -F, -O, and -OH terminations, respectively. (**D**)-(**F**) show HBs within the water region for -F, -O, and -OH terminations, respectively.

8.4 HB lifetime

On the other hand, the lifetime of a hydrogen bonds is continuously breaking and reforming, a rapid process crucial to water's dynamic nature. The HB lifetime within the confined water, between the water and MXene surface, as well as between -OH surface termination and water are all investigated and shown in Figs. S15 to S17.

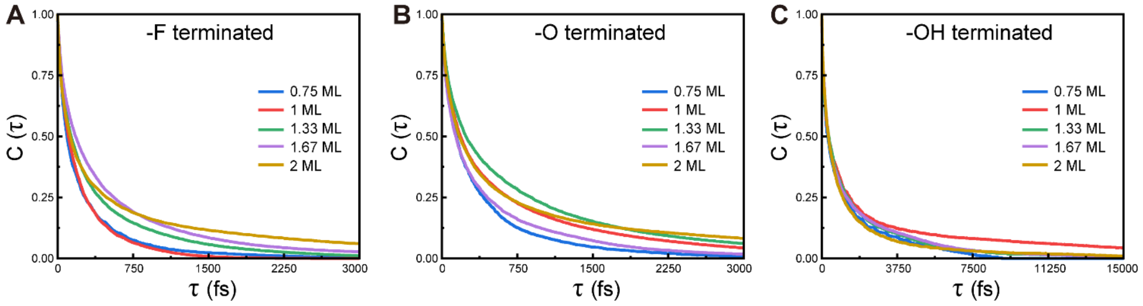

Fig. S15. HB lifetime within confined water. HB lifetime correlation functions $C_{HB}(t)$ of confined water in (**A**) $Ti_3C_2F_2$, (**B**) $Ti_3C_2O_2$ and (**C**) $Ti_3C_2(OH)_2$ MXenes.

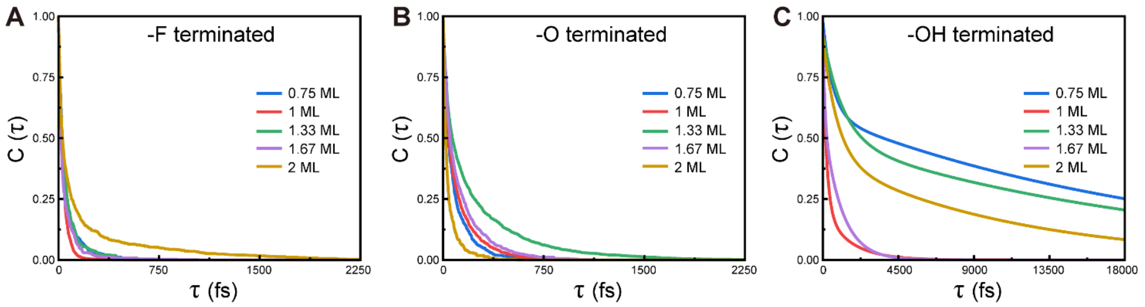

Fig. S16. HB lifetime between confined water and surface terminations. HB lifetime correlation functions $C_{HB}(t)$ established between confined water (donor) and surface terminations (acceptor) in (**A**) $Ti_3C_2F_2$, (**B**) $Ti_3C_2O_2$ and (**C**) $Ti_3C_2(OH)_2$ MXenes.

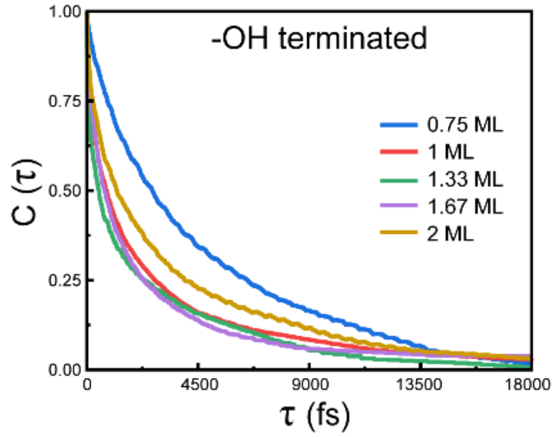

Fig. S17. HB lifetime between -OH terminations and confined water. HB lifetime correlation function $C_{HB}(t)$ established between -OH terminations (donor) and confined water (acceptor) in $Ti_3C_2(OH)_2$ MXenes.

Supplemental Text 9. Mean square displacement

Regarding the diffusivity of water, we plot the mean square displacement (MSD) of water under different surface terminations and intercalated water contents.

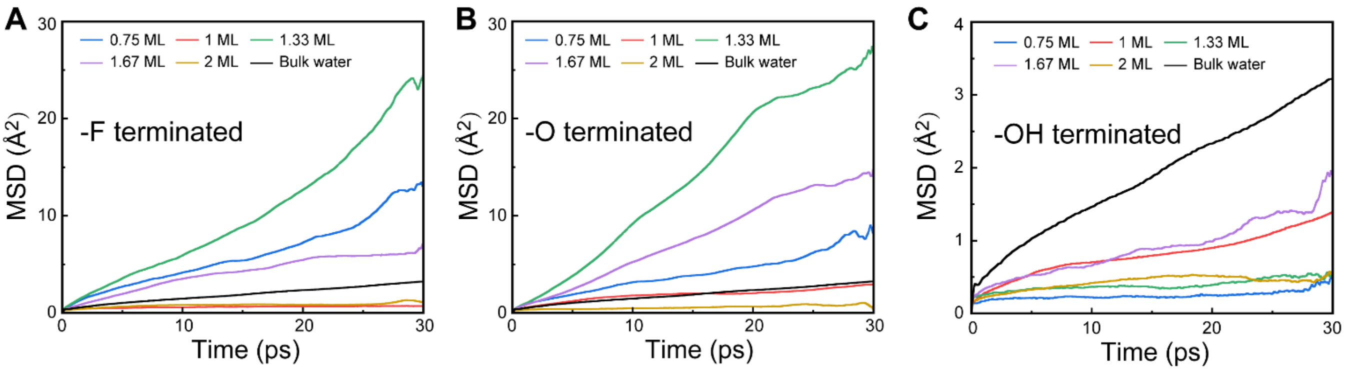

Fig. S18. Mean square displacement (MSD) calculations. MSD of confined water at varying content in (**A**) -F terminated, (**B**) -O terminated and (**C**) -OH terminated MXenes.

Supplemental Text 10. Neutron scattering spectra and zeta-potential

Inelastic (INS) and quasi-elastic neutron-scattering (QENS) experiments were performed at two neutron-scattering facilities. The INS spectra from MXene samples were obtained using the Fine Resolution Fermi Chopper Spectrometer (SEQUOIA) (*101, 102*) with incident neutron energies (E_i) of 250 meV at the Spallation Neutron Source at Oak Ridge National Laboratory. QENS experiment on the MXene (48% HF acid etched) sample was carried out at the backscattering silicon spectrometer (BASIS) (*110*) in the same facility. QENS on the MXene synthesized using 10% HF acid was performed at the National Institute of Standards and Technology (NIST) Center for Neutron Research using a high flux backscattering spectrometer (HFBS) (*111*).

The measured inelastic neutron-scattering spectra, comprising $S(Q,E)$, can be expressed as $S(Q,E) = \sum_i \frac{f_i \sigma_i \hbar^2 Q^2}{2m_i E} e^{-u_i^2 Q^2} G_i(E)[n(E,T)+1]$, where E and Q are the neutron energy and momentum transfer, respectively, and the summation runs over all atomic species i. Here, f_i, σ_i, m_i, u_i^2, and $G_i(E)$ denote the atomic fraction, neutron scattering cross-section, atomic mass, mean-squared displacement, and the partial vibrational density of states of atom i, respectively. The Bose population factor is given by $n(E,T) = [exp(E/kBT) - 1]^{-1}$. For hydrogen, the ratio σ_H / $m_H \approx 82$ barn/a.u. is more than two orders of magnitude greater than for the other constituent atoms (C, O, F, and Ti), making their contributions to $S(Q,E)$ negligible. Therefore, the generalized vibrational density of states (GVDS), $G(E)$, can be obtained from the $S(Q,E)$ spectrum as, $G(\mathrm{E}) = \int S(Q,E) \frac{EdQ}{Q^2[n(E,T)+1]}$.

The $S(Q,E)$ measured in the QENS experiment is modeled to a Lorentzian function of half width at half maximum given by Γ(Q) as (*112, 113*): $S_{QENS}(Q,E) = \frac{1}{\pi} \frac{\Gamma(Q)}{E^2+\Gamma^2(Q)}$. Q-dependence of Γ(Q) provides the nature of the self-diffusion processes. When Γ(Q) increases linearly at low Q and becomes flattened at higher Qs, the process is well described by a jump diffusion model, $\Gamma(\mathrm{Q}) = \frac{DQ^2}{1+DQ^2\tau_0}$, with the diffusion coefficient (D) and the residence time (τ_0).

Figure S19A shows the $G(E)$ spectra for 10% HF and 48% HF etched $Ti_3C_2T_x$ MXenes, measured with incident neutrons of energy E_i = 250 meV to probe the intramolecular H-O-H bending (scissors) mode of water around 205 meV, and the librational vibrations band in the 60 to 140 meV range. Both spectra exhibit a peak at 205 meV, confirming the presence of water molecules in both samples. After

normalizing the spectra to the same integrated area (i.e., approximately the same hydrogen content), the 48% HF etched MXene shows a significantly higher intensity (~1.2 ×) in the H-O-H bending region (E=205±20 meV) and a lower intensity (~1.14 ×) in the librational region compared to the 10% HF etched MXene. The later also displays a distinct peak near 120 meV, characteristic of the deformation mode of -OH groups. These features indicate a higher concentration of -OH surface terminations in the 10% HF etched MXene. Similar results were reported by Wang *et al.* (*114*), who quantitatively analyzed the surface composition and determined -OH fraction of 0.52 for the 10% HF-etched sample and 0.42 for the 48% HF-etched sample. Accordingly, the 10% HF-etched sample can be described as hydroxyl-rich $Ti_3C_2T_x$ and the 48% HF-etched sample is hydroxyl-poor $Ti_3C_2T_x$, as indicated in Fig. S19A.

Long-range translational self-diffusivity of confined water in MXenes with different -OH fractions is presented based on the dependence of the width of the QENS signals with Q^2 (more details are found in references (*37, 44, 115*)) in Fig. S19B. Self-diffusion coefficient of water in the hydroxyl-poor MXene (bottom panel) is 1.10×10^{-9} m^2s^{-1} compared to the value of 6.97×10^{-12} m^2s^{-1} (top panel) obtained from the hydroxyl-rich MXene. Even though the water molecules in the hydroxyl-poor MXene are found to reside in the inter-stack gaps (*44*), the two-order-of-magnitude reduction in water self-diffusivity relates to the higher amount of -OH groups present on the hydroxyl-rich MXene as illustrated from the INS spectra. A higher number of the -OH groups makes the surface more hydrophilic, resulting in a more robust interaction with water molecules, thereby reducing the translational mobility of water molecules. The surface-dependent diffusion behavior enhances the qualitative agreement between simulations and experiments.

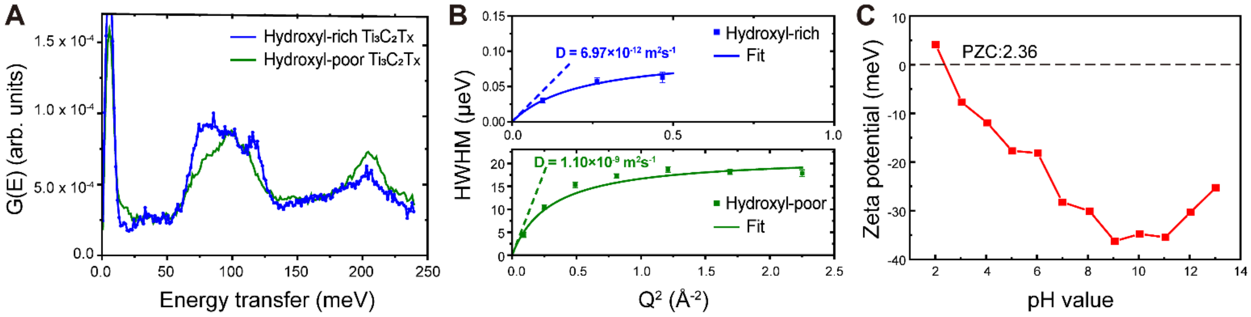

Fig. S19. Experimental validation using neutron scattering. (**A**) GVDS or G(E) measured from MXenes synthesized using 10% HF and 48% HF using SEQUOIA with Ei = 250 meV at T = 7 K. The 10% HF-etched and 48% HF-etched samples are labeled as hydroxyl-rich (blue line) and hydroxyl-poor (green line) $Ti_3C_2T_x$, respectively. (**B**) The dependence of HWHM on Q2 is shown in two panels: the top panel depicts hydroxyl-rich $Ti_3C_2T_x$, and the bottom panel depicts hydroxyl-poor $Ti_3C_2T_x$. The solid lines in both panels represent the fit to the jump-diffusion model, while the dotted lines

represent the slopes that evaluate the diffusion coefficients. Data in (A,B) adopted from the references (*37, 44, 115*) and replotted. (**C**) Zeta potential of 0.1 mg mL^{-1} $Ti_3C_2T_x$-10% suspension depends on pH. Data adopted from the reference (*116*) and replotted.

Supplemental Text 11. Formation energy of the functionalized MXenes

In general, the formation energy of the functionalized MXene sheets is defined as the energy change during the formation of a functionalized MXene sheet from its constituent components, and can be calculated according to (*117, 118*):

$$\Delta H_f = E_{tot}(M_{n+1}X_nT_m) - E_{tot}(M_{n+1}X_n) - m \cdot \mu_T^{T_2} - m \cdot \Delta\mu_T, \quad \text{(S3)}$$

where $E_{tot}(M_{n+1}X_nT_m)$ is the energy of the MXene with T = -F, -O, or -OH terminations, $E_{tot}(M_{n+1}X_n)$ is the energy of the bare MXene, $\mu_T^{T_2}$ is the energy of the terminating atoms in their gaseous reference state ($T_2 = F_2$, O_2, or $O_2 + H_2$), and $\Delta\mu_T = \mu_T^{liquid} - \mu_T^{T_2}$ represents the difference in chemical potential, where μ_T^{liquid} is the chemical potential of the terminating atoms in the liquid phase. Here, we consider the favorability of surface terminations in the aqueous environment, aligned with the experimental access via the wet etching process according to the following reactions:

$$Ti_3C_2 + 2HF \rightarrow Ti_3C_2F_2 + H_2, \quad \text{(S4)}$$

$$Ti_3C_2 + 2H_2O \rightarrow Ti_3C_2O_2 + 2H_2, \quad \text{(S5)}$$

$$Ti_3C_2 + 2H_2O \rightarrow Ti_3C_2(OH)_2 + H_2. \quad \text{(S6)}$$

To address liquid-phase reactions, it is necessary to define the change of chemical potentials of $\Delta\mu_T$, i.e., $\Delta\mu_F = \mu_F^{HF} - \mu_F^{F_2}$, $\Delta\mu_O = \mu_O^{H_2O} - \mu_O^{O_2}$, and $\Delta\mu_{OH} = \mu_{HO}^{H_2O} - \mu_O^{O_2} - \mu_H^{H_2}$. These relative chemical potentials are interdependent on one another through the relative chemical potential of hydrogen, as defined by $\Delta\mu_H = \mu_H^{H_2O} - \mu_H^{H_2}$.

In their thermal equilibrium states with the solution, μ_T^{liquid} can be also related with $\mu_T^{T_2}$, by the formation energy of HF and H_2O, i.e., ΔH_f^{HF} and $\Delta H_f^{H_2O}$, respectively:

$$E_{tot}(HF) = \mu_F^{HF} + \mu_H^{HF} = \mu_F^{F_2} + \mu_H^{H_2} + \Delta H_f^{HF}, \quad \text{(S7)}$$

$$E_{tot}(H_2O) = \mu_O^{H_2O} + 2\mu_H^{H_2O} = \mu_O^{O_2} + 2\mu_H^{H_2} + \Delta H_f^{H_2O}, \quad \text{(S8)}$$

$$E_{tot}(H_2O) = \mu_{HO}^{H_2O} + \mu_H^{H_2O} = \mu_O^{O_2} + 2\mu_H^{H_2} + \Delta H_f^{H_2O}, \quad \text{(S9)}$$

where $E_{tot}(HF)$ and $E_{tot}(H_2O)$ are the total energies of the HF and water molecules, respectively. By the definition of $\Delta\mu_T$, we can thus have:

$$\Delta\mu_F = \Delta H_f^{HF} - \Delta\mu_H, \quad \text{(S10)}$$

$$\Delta\mu_O = \Delta H_f^{H_2O} - 2\Delta\mu_H, \quad \text{(S11)}$$

$$\Delta\mu_{OH} = \Delta H_f^{H_2O} - \Delta\mu_H. \quad \text{(S12)}$$

For given stable surface terminations, the chemical potential of F, O, OH or H at the interface must be constrained to a condition of lower than that in its pure bulk form, meaning that $\Delta\mu_F \leq 0$, $\Delta\mu_O \leq 0$, $\Delta\mu_{OH} \leq 0$, and $\Delta\mu_H \leq 0$. Combining this condition with Eqs. (S10-S12), we can determine the following range of $\Delta\mu_H$ for each surface termination:

$$\text{-F terminations: } \Delta H_f^{HF} \leq \Delta\mu_H \leq 0, \quad \text{(S13)}$$

$$\text{-O terminations: } \frac{1}{2}\Delta H_f^{H_2O} \leq \Delta\mu_H \leq 0, \quad \text{(S14)}$$

$$\text{-OH terminations: } \Delta H_f^{H_2O} \leq \Delta\mu_H \leq 0. \quad \text{(S15)}$$

Further substituting Eqs. (S10-S12) to Eq. (S3), one can rewrite the formation energy for the three terminations specifically:

$$\Delta H_f = E_{tot}(Ti_3C_2F_2) - E_{tot}(Ti_3C_2) - 2\mu_F^{F_2} - 2\Delta H_f^{HF} + 2\Delta\mu_H, \quad \text{(S16)}$$

$$\Delta H_f = E_{tot}(Ti_3C_2O_2) - E_{tot}(Ti_3C_2) - 2\mu_O^{O_2} - 2\Delta H_f^{H_2O} + 4\Delta\mu_H, \quad \text{(S17)}$$

$$\Delta H_f = E_{tot}(Ti_3C_2(OH)_2) - E_{tot}(Ti_3C_2) - 2(\mu_O^{O_2} + \mu_H^{H_2}) - 2\Delta H_f^{H_2O} + 2\Delta\mu_H. \quad \text{(S18)}$$

These equations allow us to evaluate the formation energy of the functionalized MXene sheets. Within this framework, formation energies of the bulk HF and H_2O phases are calculated to be -2.74 eV and -2.52 eV per formula, respectively, showing good quantitative agreement with experimental measurements of ΔH_f^{HF} = -2.81 eV and $\Delta H_f^{H_2O}$ = -2.51 eV (*119-121*). By applying Eqs. (S13-S18) to the $\Delta\mu_H$-dependent formation energies, we can clearly find that the formation energy of the three different $Ti_3C_2T_2$ surface terminations increases with the increasing $\Delta\mu_H$ as shown in Fig. S20.

The surface Pourbaix diagrams, depicting the relationship between pH, applied electrode potential, and stability, were generated using the computational hydrogen electrode (CHE) approach, presented in

Fig. S21A. It offers a more accessible interpretation compared to the results with the chemical potential shown in Fig. S20. They reveal that the Ti_3C_2 surface with -O termination remains stable above the HER equilibrium line. Below this line, the -OH termination becomes more favorable due to proton reduction, while the -F termination is stable only within a narrow range near the HER line, specifically under strongly acidic conditions (pH⩽2). It is noteworthy that maintaining stability for the bare surface proves to be exceedingly challenging. Our findings align closely with those reported in Ref. (*79*) as a comparison in Fig. S21B. The discrepancy might be originated from differences in methodology, as the referenced results are based on a slab model of $Ti_3C_2T_1$, whereas our calculations utilize a stoichiometric model of $Ti_3C_2T_2$.

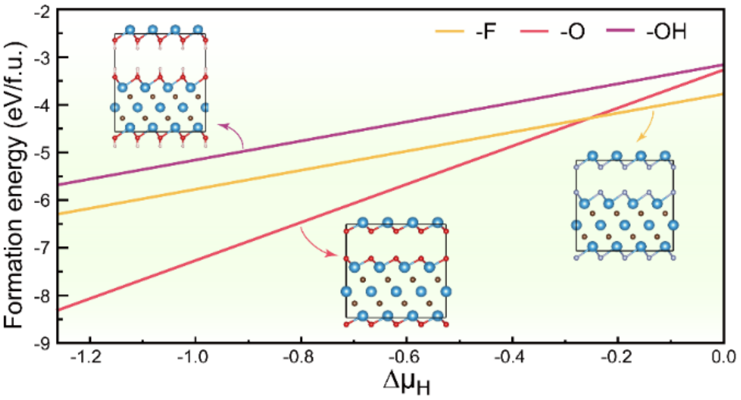

Fig. S20. Thermodynamic stability evaluation of surface groups. Formation energy *vs* $\Delta\mu_H$ for $Ti_3C_2T_2$ MXenes with terminations of T = -F, -O and -OH.

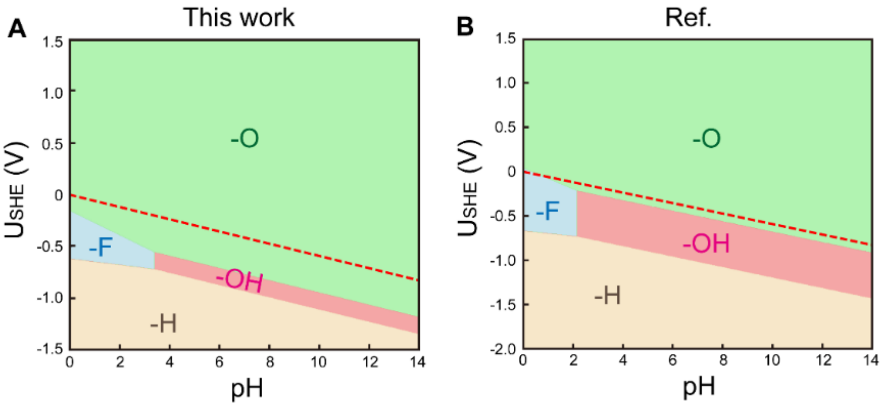

Fig. S21. Surface Pourbaix diagrams. Calculated Pourbaix diagrams for $Ti_3C_2T_2$ MXene with -F, -O, -OH, and -H terminations: (**A**) results obtained in this work; (**B**) reference results (*79*).

Supplemental Text 12. θ_n proportion

The orientation parameter θ_n captures both the individual and collective alignment of water molecules driven by electrostatic and hydrogen-bond interactions, and its statistical distribution is shown in Fig. S22.

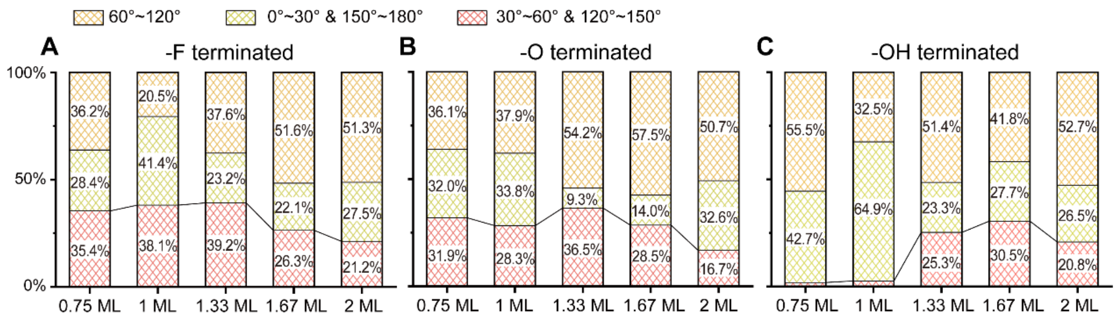

Fig. S22. Statistical analysis of water molecule orientations. The proportion of θ_n at (**A**) -F terminated, (**B**) -O terminated and (**C**) -OH terminated interfaces. $\theta_n(\perp)$ = 60°–120° corresponds to H_2O molecules perpendicular to the $Ti_3C_2T_2$ surface, $\theta_n(\parallel)$ = 0°–30° & 150°–180° corresponds to H_2O molecules parallel to the $Ti_3C_2T_2$ surface and $\theta_n(\parallel \rightleftharpoons \perp)$ = 30°–60° & 120°–150° represents H_2O molecules in an intermediate state.

Supplemental Text 13. Effects of halogen and mixed terminations on confined water

Since halogen terminations, e.g. -Cl achieved through the molten salt method, are known to influence surface chemistry and interfacial interactions, we also discuss the effect of -Cl termination on the surface chemistry and solid-liquid interface in Fig. S23. Moreover, the influence of mixed surface terminations, as more representative of experimentally synthesized $Ti_3C_2T_x$ MXenes, was also investigated and exemplified by the combination of -Cl and -O, as well as -O and -OH, as illustrated in Fig. S24.

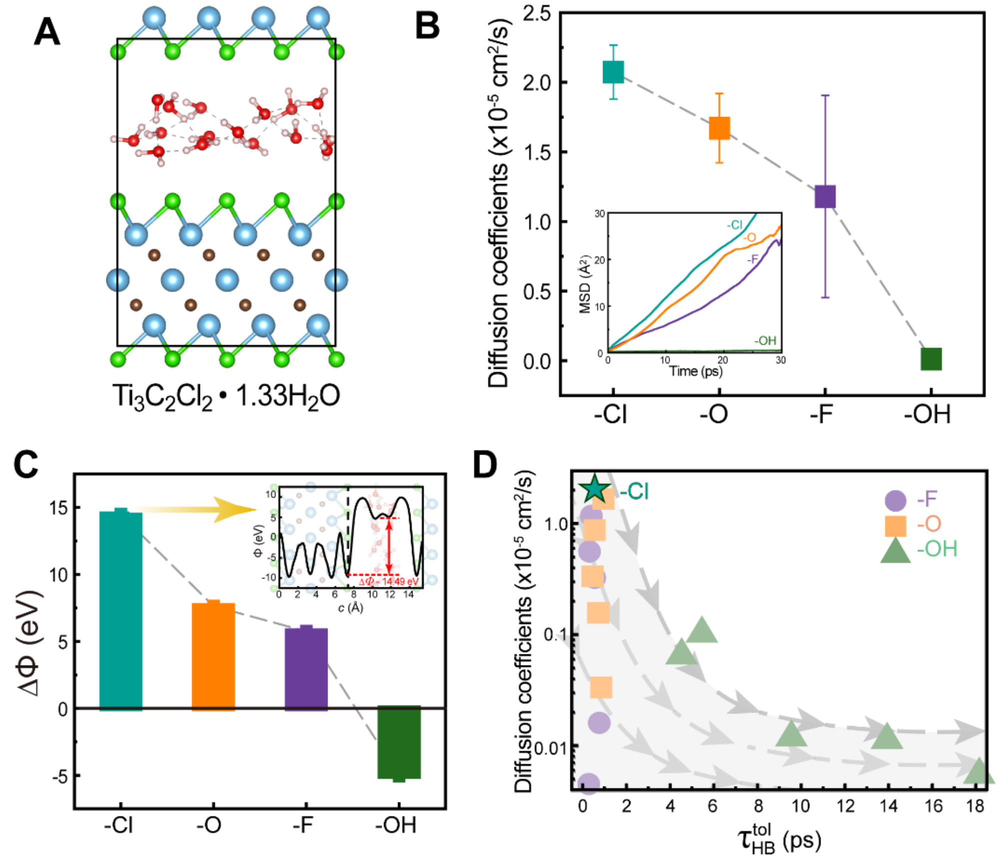

Fig. S23. Effects of -Cl terminations on confined water. (**A**) Interface model with -Cl terminations and 1.33 ML water,

exhibiting an interlayer spacing of 15.4 Å. (**B**) Diffusion coefficients of confined water at different terminated interfaces. (**C**) The electrostatic potential difference ΔΦ at different terminated interface. (**D**) Diffusion coefficients versus total hydrogen-bond lifetimes τ_{HB}^{tol}.

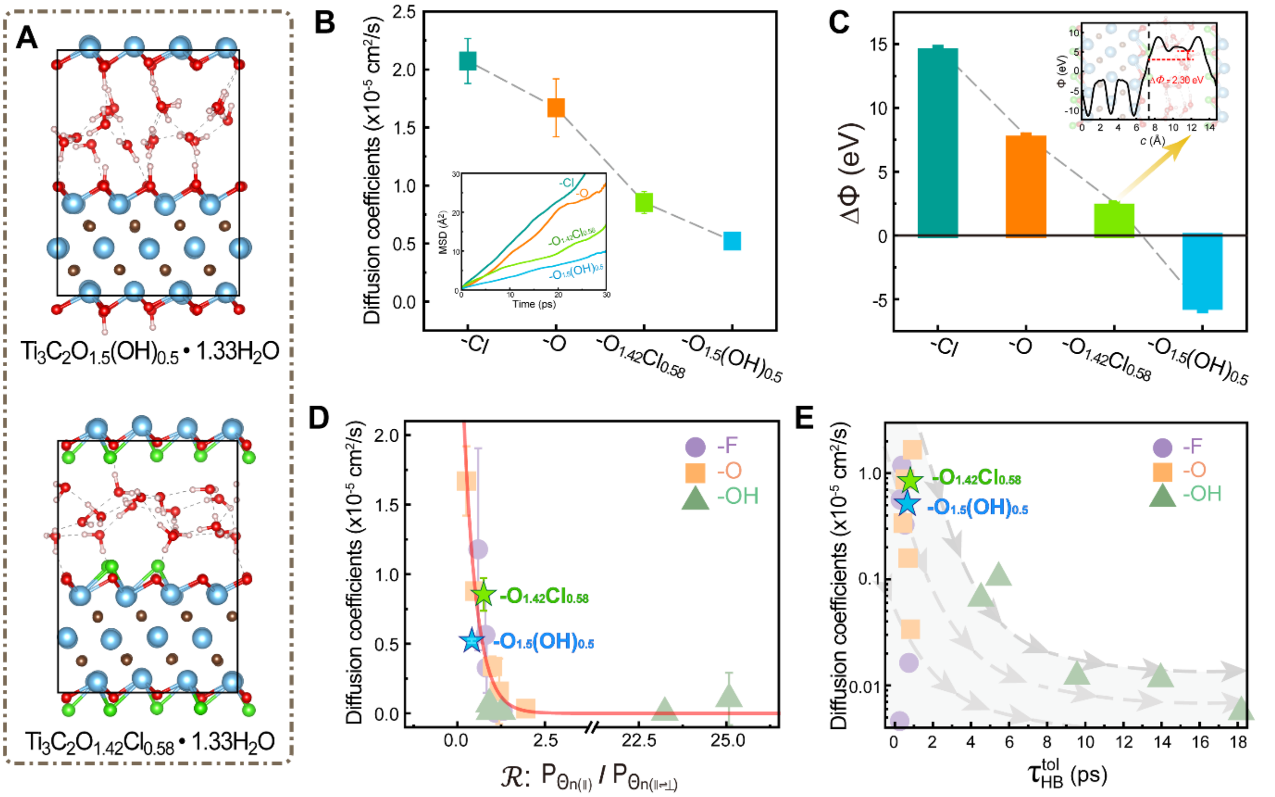

Fig. S24. Effects of mixed terminations on confined water. (**A**) Interface model with mixed terminations and 1.33 ML water, showing interlayer spacings of 14.3 Å and 14.8 Å for $Ti_3C_2O_{1.5}(OH)_{0.5}$ and $Ti_3C_2O_{1.42}Cl_{0.58}$. (**B**) Diffusion coefficients of confined water at different terminated interfaces. (**C**) The electrostatic potential difference ΔΦ at different terminated interface. (**D**) The relationship between diffusion coefficients and the orientational ratio $\mathcal{R}$ of $P_{\theta n(\parallel)}$ to $P_{\theta n(\parallel\rightleftharpoons\perp)}$. $P_{\theta n(\parallel)}$ and $P_{\theta n(\parallel\rightleftharpoons\perp)}$ represent the proportion of water molecules with a normal angle of 0°–30° & 150°–180° and 30°–60° & 120°–150°, respectively. (**E**) Diffusion coefficients versus total hydrogen-bond lifetimes τ_{HB}^{tol}.

Note: Certain commercial material suppliers are identified in this paper to foster understanding. Such identification does not imply recommendation or endorsement by the NIST, nor does it imply that the materials or equipment identified are necessarily the best available for the purpose.

REFERENCES

1. Y. Xu, Nanofluidics: A new arena for materials science. *Adv. Mater.* **30**, 1702419 (2018).

2. D. Wang, Y. Tian, L. Jiang, Abnormal properties of low-dimensional confined water. *Small* **17**, e2100788 (2021).

3. J. K. Holt, H. G. Park, Y. Wang, M. Stadermann, A. B. Artyukhin, C. P. Grigoropoulos, A. Noy, O. Bakajin, Fast mass transport through sub-2-nanometer carbon nanotubes. *Science* **312**, 1034–1037 (2006).

4. D. Takaiwa, I. Hatano, K. Koga, H. Tanaka, Phase diagram of water in carbon nanotubes. *Proc. Natl. Acad. Sci. U.S.A.* **105**, 39–43 (2008).

5. M. Antognozzi, A. D. L. Humphris, M. J. Miles, Observation of molecular layering in a confined water film and study of the layers viscoelastic properties. *Appl. Phys. Lett.* **78**, 300–302 (2001).

6. L. Fumagalli, A. Esfandiar, R. Fabregas, S. Hu, P. Ares, A. Janardanan, Q. Yang, B. Radha, T. Taniguchi, K. Watanabe, G. Gomila, K. S. Novoselov, A. K. Geim, Anomalously low dielectric constant of confined water. *Science* **360**, 1339–1342 (2018).

7. N. Giovambattista, P. J. Rossky, P. G. Debenedetti, Computational studies of pressure, temperature, and surface effects on the structure and thermodynamics of confined water. *Annu. Rev. Phys. Chem.* **63**, 179–200 (2012).

8. M. Favaro, B. Jeong, P. N. Ross, J. Yano, Z. Hussain, Z. Liu, E. J. Crumlin, Unravelling the electrochemical double layer by direct probing of the solid/liquid interface. *Nat. Commun.* **7**, 12695 (2016).

9. Z. Zhang, X. Li, J. Yin, Y. Xu, W. Fei, M. Xue, Q. Wang, J. Zhou, W. Guo, Emerging hydrovoltaic technology. *Nat. Nanotechnol.* **13**, 1109–1119 (2018).

10. J. Fritz, M. K. Baller, H. P. Lang, T. Strunz, E. Meyer, H. J. Güntherodt, E. Delamarche, C. Gerber, J. K. Gimzewski, Stress at the solid-liquid interface of self-assembled monolayers on gold investigated with a nanomechanical sensor. *Langmuir* **16**, 9694–9696 (2000).

11. J. Carrasco, A. Hodgson, A. Michaelides, A molecular perspective of water at metal interfaces. *Nat. Mater.* **11**, 667–674 (2012).

12. D. Muñoz-Santiburcio, D. Marx, Confinement-controlled aqueous chemistry within nanometric slit pores. *Chem. Rev.* **121**, 6293–6320 (2021).

13. Q. Li, J. Song, F. Besenbacher, M. Dong, Two-dimensional material confined water. *Acc. Chem. Res.* **48**, 119–127 (2014).

14. G. Hummer, J. C. Rasaiah, J. P. Noworyta, Water conduction through the hydrophobic channel of a carbon nanotube. *Nature* **414**, 188–190 (2001).

15. M. Majumder, N. Chopra, R. Andrews, B. J. Hinds, Enhanced flow in carbon nanotubes. *Nature* **438**, 44–44 (2005).

16. B. Radha, A. Esfandiar, F. C. Wang, A. P. Rooney, K. Gopinadhan, A. Keerthi, A. Mishchenko, A. Janardanan, P. Blake, L. Fumagalli, M. Lozada-Hidalgo, S. Garaj, S. J. Haigh, I. V. Grigorieva, H. A. Wu, A. K. Geim, Molecular transport through capillaries made with atomic-scale precision. *Nature* **538**, 222–225 (2016).

17. R. Devanathan, D. Chase-Woods, Y. Shin, D. W. Gotthold, Molecular dynamics simulations reveal that water diffusion between graphene oxide layers is slow. *Sci. Rep.* **6**, 29484 (2016).

18. Z. Wang, Q. Tu, S. Zheng, J. J. Urban, S. Li, B. Mi, Understanding the aqueous stability and filtration capability of MoS_2 membranes. *Nano Lett.* **17**, 7289–7298 (2017).

19. C. Chen, J. Wang, D. Liu, C. Yang, Y. Liu, R. S. Ruoff, W. Lei, Functionalized boron nitride membranes with ultrafast solvent transport performance for molecular separation. *Nat. Commun.* **9**, 1902 (2018).

20. M. Naguib, M. Kurtoglu, V. Presser, J. Lu, J. Niu, M. Heon, L. Hultman, Y. Gogotsi, M. W. Barsoum, Two-dimensional nanocrystals produced by exfoliation of Ti_3AlC_2. *Adv. Mater.* **23**, 4248–4253 (2011).

21. M. Li, J. Lu, K. Luo, Y. Li, K. Chang, K. Chen, J. Zhou, J. Rosen, L. Hultman, P. Eklund, P. O. A. Persson, S. Du, Z. Chai, Z. Huang, Q. Huang, Element replacement approach by reaction with lewis acidic molten salts to synthesize nanolaminated MAX phases and MXenes. *J. Am. Chem. Soc.* **141**, 4730–4737 (2019).

22. Y. Li, H. Shao, Z. Lin, J. Lu, L. Liu, B. Duployer, P. O. Å. Persson, P. Eklund, L. Hultman, M. Li, K. Chen, X.-H. Zha, S. Du, P. Rozier, Z. Chai, E. Raymundo-Piñero, P.-L. Taberna, P. Simon, Q. Huang, A general Lewis acidic etching route for preparing MXenes with enhanced electrochemical performance in non-aqueous electrolyte. *Nat. Mater.* **19**, 894–899 (2020).

23. V. Kamysbayev, A. S. Filatov, H. Hu, X. Rui, F. Lagunas, D. Wang, R. F. Klie, D. V. Talapin, Covalent surface modifications and superconductivity of two-dimensional metal carbide MXenes. *Science* **369**, 979–983 (2020).

24. A. Sugahara, Y. Ando, S. Kajiyama, K. Yazawa, K. Gotoh, M. Otani, M. Okubo, A. Yamada, Negative dielectric constant of water confined in nanosheets. *Nat. Commun.* **10**, 850 (2019).

25. Y. Sun, C. Zhan, P. R. C. Kent, M. Naguib, Y. Gogotsi, D.-E. Jiang, Proton redox and transport in MXene-confined water. *ACS Appl. Mater. Interfaces* **12**, 763–770 (2020).

26. L. Liu, E. Raymundo-Piñero, S. Sunny, P.-L. Taberna, P. Simon, Role of surface terminations for charge storage of $Ti_3C_2T_x$ MXene electrodes in aqueous acidic electrolyte. *Angew. Chem. Int. Ed. Engl.* **63**, e202319238 (2024).

27. M. R. Lukatskaya, O. Mashtalir, C. E. Ren, Y. Dall'Agnese, P. Rozier, P. L. Taberna, M. Naguib, P. Simon, M. W. Barsoum, Y. Gogotsi, Cation intercalation and high volumetric capacitance of two-dimensional titanium carbide. *Science* **341**, 1502–1505 (2013).

28. M. Naguib, V. N. Mochalin, M. W. Barsoum, Y. Gogotsi, 25th anniversary article: MXenes: A new family of two-dimensional materials. *Adv. Mater.* **26**, 992–1005 (2014).

29. B. Anasori, M. R. Lukatskaya, Y. Gogotsi, 2D metal carbides and nitrides (MXenes) for energy storage. *Nat. Rev. Mater.* **2**, 16098 (2017).

30. M. Hu, T. Hu, Z. Li, Y. Yang, R. Cheng, J. Yang, C. Cui, X. Wang, Surface functional groups and interlayer water determine the electrochemical capacitance of $Ti_3C_2T_x$ MXene. *ACS Nano* **15**, 7835–7835 (2021).

31. H. Li, K. Xu, P. Chen, Y. Yuan, Y. Qiu, L. Wang, L. Zhu, X. Wang, G. Cai, L. Zheng, C. Dai, D. Zhou, N. Zhang, J. Zhu, J. Xie, F. Liao, H. Peng, Y. Peng, J. Ju, Z. Lin, J. Sun, Achieving ultrahigh electrochemical performance by surface design and nanoconfined water manipulation. *Natl. Sci. Rev.* **9**, nwac079 (2022).

32. H. Shao, K. Xu, Y.-C. Wu, A. Iadecola, L. Liu, H. Ma, L. Qu, E. Raymundo-Pinero, J. Zhu, Z. Lin, P.-L. Taberna, P. Simon, Unraveling the charge storage mechanism of $Ti_3C_2T_x$ MXene electrode in acidic electrolyte. *ACS Energy Lett.* **5**, 2873–2880 (2020).

33. M. Okubo, A. Sugahara, S. Kajiyama, A. Yamada, Mxene as a charge storage host. *Acc. Chem. Res.* **51**, 591–599 (2018).

34. Y. Ando, M. Okubo, A. Yamada, M. Otani, Capacitive versus pseudocapacitive storage in MXene. *Adv. Funct. Mater.* **30**, 2000820 (2020).

35. Q. Gao, W. Sun, P. Ilani-Kashkouli, A. Tselev, P. R. C. Kent, N. Kabengi, M. Naguib, M. Alhabeb, W.-Y. Tsai, A. P. Baddorf, J. Huang, S. Jesse, Y. Gogotsi, N. Balke, Tracking ion intercalation into layered Ti_3C_2 MXene films across length scales. *Energ. Environ. Sci.* **13**, 2549–2558 (2020).

36. N. Shpigel, M. D. Levi, S. Sigalov, T. S. Mathis, Y. Gogotsi, D. Aurbach, Direct assessment of nanoconfined water in 2D Ti_3C_2 electrode interspaces by a surface acoustic technique. *J. Am. Chem. Soc.* **140**, 8910–8917 (2018).

37. N. C. Osti, M. Naguib, K. Ganeshan, Y. K. Shin, A. Ostadhossein, A. C. T. van Duin, Y. Cheng, L. L. Daemen, Y. Gogotsi, E. Mamontov, A. I. Kolesnikov, Influence of metal ions intercalation on the vibrational dynamics of water confined between MXene layers. *Phys. Rev. Mater.* **1**, 065406 (2017).

38. M. L. Martins, T. S. Mathis, X. Wang, L. L. Daemen, Y. Gogotsi, E. Mamontov, Water dynamics in pristine and porous $Ti_3C_2T_x$ MXene as probed by quasielastic neutron scattering. *Phys. Rev. Mater.* **6**, 034001 (2022).

39. P. Hou, Y. Tian, Y. Xie, Q. Li, G. Chen, F. Du, J. Wu, Y. Ma, X. Meng, Proton-driven dynamic behavior of nanoconfined water in hydrophilic MXene sheets. *Angew. Chem. Int. Ed. Engl.* **63**, e202411849 (2024).

40. R. Jinnouchi, F. Karsai, G. Kresse, On-the-fly machine learning force field generation: Application to melting points. *Phys. Rev. B* **100**, 014105 (2019).

41. J. Yang, M. Li, S. Fang, Y. Wang, H. He, C. Wang, Z. Zhang, B. Yuan, L. Jiang, R. H. Baughman, Q. Cheng, Water-induced strong isotropic MXene-bridged graphene sheets for electrochemical energy storage. *Science* **383**, 771–777 (2024).

42. S. Vaitheeswaran, J. C. Rasaiah, G. Hummer, Electric field and temperature effects on water in the narrow nonpolar pores of carbon nanotubes. *J. Chem. Phys.* **121**, 7955–7965 (2004).

43. X. Mu, D. Wang, F. Du, G. Chen, C. Wang, Y. Wei, Y. Gogotsi, Y. Gao, Y. Dall'Agnese, Revealing the pseudo-intercalation charge storage mechanism of MXenes in acidic electrolyte. *Adv. Funct. Mater.* **29**, 1902953 (2019).

44. N. C. Osti, M. Naguib, A. Ostadhossein, Y. Xie, P. R. C. Kent, B. Dyatkin, G. Rother, W. T. Heller, A. C. T. van Duin, Y. Gogotsi, E. Mamontov, Effect of metal ion intercalation on the structure of MXene and water dynamics on its internal surfaces. *ACS Appl. Mater. Interfaces* **8**, 8859–8863 (2016).

45. R. Cheng, T. Hu, H. Zhang, C. Wang, M. Hu, J. Yang, C. Cui, T. Guang, C. Li, C. Shi, P. Hou, X. Wang, Understanding the lithium storage mechanism of $Ti_3C_2T_x$ MXene. *J. Phys. Chem. C* **123**, 1099–1109 (2019).

46. Y. Xie, M. Naguib, V. N. Mochalin, M. W. Barsoum, Y. Gogotsi, X. Yu, K.-W. Nam, X.-Q. Yang, A. I. Kolesnikov, P. R. C. Kent, Role of surface structure on Li-ion energy storage capacity of two-dimensional transition-metal carbides. *J. Am. Chem. Soc.* **136**, 6385–6394 (2014).

47. F. Chang, C. Li, J. Yang, H. Tang, M. Xue, Synthesis of a new graphene-like transition metal carbide by de-intercalating Ti_3AlC_2. *Mater. Lett.* **109**, 295–298 (2013).

48. M. Naguib, O. Mashtalir, J. Carle, V. Presser, J. Lu, L. Hultman, Y. Gogotsi, M. W. Barsoum, Two-dimensional transition metal carbides. *ACS Nano* **6**, 1322–1331 (2012).

49. M. Ghidiu, S. Kota, V. Drozd, M. W. Barsoum, Pressure-induced shear and interlayer expansion in Ti_3C_2 MXene in the presence of water. *Sci. Adv.* **4**, eaao6850 (2018).

50. C. Zhan, M. Naguib, M. Lukatskaya, P. R. C. Kent, Y. Gogotsi, D.-E. Jiang, Understanding the MXene pseudocapacitance. *J. Phys. Chem. Lett.* **9**, 1223–1228 (2018).

51. T. Kobayashi, Y. Sun, K. Prenger, D.-E. Jiang, M. Naguib, M. Pruski, Nature of terminating hydroxyl groups and intercalating water in $Ti_3C_2T_x$ MXenes: A study by ^{1}H solid-state NMR and DFT calculations. *J. Phys. Chem. C* **124**, 13649–13655 (2020).

52. T. S. Mathis, K. Maleski, A. Goad, A. Sarycheva, M. Anayee, A. C. Foucher, K. Hantanasirisakul, C. E. Shuck, E. A. Stach, Y. Gogotsi, Modified MAX phase synthesis for environmentally stable and highly conductive Ti_3C_2 MXene. *ACS Nano* **15**, 6420–6429 (2021).

53. M. Ghidiu, J. Halim, S. Kota, D. Bish, Y. Gogotsi, M. W. Barsourm, Ion-exchange and cation solvation reactions in Ti_3C_2 MXene. *Chem. Mater.* **28**, 3507–3514 (2016).

54. D. Wu, Z. Zhao, B. Lin, Y. Song, J. Qi, J. Jiang, Z. Yuan, B. Cheng, M. Zhao, Y. Tian, Z. Wang, M. Wu, K. Bian, K.-H. Liu, L.-M. Xu, X. C. Zeng, E.-G. Wang, Y. Jiang, Probing structural superlubricity of two-dimensional water transport with atomic resolution. *Science* **384**, 1254–1259 (2024).

55. Y. Wang, F. Tang, X. Yu, K.-Y. Chiang, C.-C. Yu, T. Ohto, Y. Chen, Y. Nagata, M. Bonn, Interfaces govern the structure of angstrom-scale confined water solutions. *Nat. Commun.* **16**, 7288 (2025).

56. J. P. Straley, Ordered phases of a liquid of biaxial particles. *Phys. Rev. A* **10**, 1881–1887 (1974).

57. D. K. Hore, D. S. Walker, G. L. Richmond, Water at hydrophobic surfaces: When weaker is better. *J. Am. Chem. Soc.* **130**, 1800–1801 (2008).

58. G. Algara-Siller, O. Lehtinen, F. C. Wang, R. R. Nair, U. Kaiser, H. A. Wu, A. K. Geim, I. V. Grigorieva, Square ice in graphene nanocapillaries. *Nature* **519**, 443–445 (2015).

59. K. M. Herman, S. S. Xantheas, Origins of tetrahedral order in ice. *J. Am. Chem. Soc.* **147**, 29732–29741 (2025).

60. S. Varghese, S. K. Kannam, J. S. Hansen, S. P. Sathian, Effect of hydrogen bonds on the dielectric properties of interfacial water. *Langmuir* **35**, 8159–8166 (2019).

61. R. Walker-Gibbons, A. Kubincová, P. H. Hünenberger, M. Krishnan, The role of surface chemistry in the orientational behavior of water at an interface. *J. Phys. Chem. B* **126**, 4697–4710 (2022).

62. E. H. Thiede, B. Van Koten, J. Weare, A. R. Dinner, Eigenvector method for umbrella sampling enables error analysis. *J. Chem. Phys.* **145**, 084115 (2016).

63. T. Dufils, C. Schran, J. Chen, A. K. Geim, L. Fumagalli, A. Michaelides, Origin of dielectric polarization suppression in confined water from first principles. *Chem. Sci.* **15**, 516–527 (2024).

64. J.-B. Le, Q.-Y. Fan, J.-Q. Li, J. Cheng, Molecular origin of negative component of Helmholtz capacitance at electrified Pt(111)/water interface. *Sci. Adv.* **6**, eabb1219 (2020).

65. J. Cheng, M. Sprik, Alignment of electronic energy levels at electrochemical interfaces. *Phys. Chem. Chem. Phys.* **14**, 11245–11267 (2012).

66. D. C. Rapaport, Hydrogen bonds in water. *Mol. Phys.* **50**, 1151–1162 (1983).

67. R. J. Gowers, P. Carbone, A multiscale approach to model hydrogen bonding: The case of polyamide. *J. Chem. Phys.* **142**, 224907 (2015).

68. A. Luzar, D. Chandler, Structure and hydrogen bond dynamics of water-dimethyl sulfoxide mixtures by computer simulations. *J. Chem. Phys.* **98**, 8160–8173 (1993).

69. S. Park, M. D. Fayer, Hydrogen bond dynamics in aqueous NaBr solutions. *Proc. Natl. Acad. Sci. U.S.A.* **104**, 16731–16738 (2007).

70. M. L. Antipova, V. E. Petrenko, Hydrogen bond lifetime for water in classic and quantum molecular dynamics. *Russ. J. Phys. Chem. A* **87**, 1170–1174 (2013).

71. I. Hanasaki, A. Nakatani, Hydrogen bond dynamics and microscopic structure of confined water inside carbon nanotubes. *J. Chem. Phys.* **124**, 174714 (2006).

72. C.-C. Yu, K.-Y. Chiang, M. Okuno, T. Seki, T. Ohto, X. Yu, V. Korepanov, H.-o. Hamaguchi, M. Bonn, J. Hunger, Y. Nagata, Vibrational couplings and energy transfer pathways of water's bending mode. *Nat. Commun.* **11**, 5977 (2020).

73. M. Flór, D. M. Wilkins, M. de la Puente, D. Laage, G. Cassone, A. Hassanali, S. Roke, Dissecting the hydrogen bond network of water: Charge transfer and nuclear quantum effects. *Science* **386**, eads4369 (2024).

74. M. Chen, H. Y. Ko, R. C. Remsing, M. F. Calegari Andrade, B. Santra, Z. Sun, A. Selloni, R. Car, M. L. Klein, J. P. Perdew, X. Wu, Ab initio theory and modeling of water. *Proc. Natl. Acad. Sci. U.S.A.* **114**, 10846–10851 (2017).

75. J. Li, Y. Lin, T. Meier, Z. Liu, W. Yang, H.-k. Mao, S. Zhu, Q. Hu, Silica-water superstructure and one-dimensional superionic conduit in Earth's mantle. *Sci. Adv.* **9**, eadh3784 (2023).

76. A. Gentile, C. Ferrara, S. Tosoni, M. Balordi, S. Marchionna, F. Cernuschi, M.-H. Kim, H.-W. Lee, R. Ruffo, Enhanced functional properties of $Ti_3C_2T_x$ Mxenes as negative electrodes in sodium-ion batteries by chemical tuning. *Small Methods* **4**, 2000314 (2020).

77. M. Seredych, C. E. Shuck, D. Pinto, M. Alhabeb, E. Precetti, G. Deysher, B. Anasori, N. Kurra, Y. Gogotsi, High-temperature behavior and surface chemistry of carbide MXenes studied by thermal analysis. *Chem. Mater.* **31**, 3324–3332 (2019).

78. M. A. Hope, A. C. Forse, K. J. Griffith, M. R. Lukatskaya, M. Ghidiu, Y. Gogotsi, C. P. Grey, NMR reveals the surface functionalisation of Ti_3C_2 MXene. *Phys. Chem. Chem. Phys.* **18**, 5099–5102 (2016).

79. M. López, K. S. Exner, F. Viñes, F. Illas, Computational pourbaix diagrams for MXenes: A key ingredient toward proper theoretical electrocatalytic studies. *Adv. Theory Simul.* **6**, 2200217 (2023).

80. S. O. Diallo, E. Mamontov, W. Nobuo, S. Inagaki, Y. Fukushima, Enhanced translational diffusion of confined water under electric field. *Phys. Rev. E* **86**, 021506 (2012).

81. M. Sobrino Fernández, F. M. Peeters, M. Neek-Amal, Electric-field-induced structural changes in water confined between two graphene layers. *Phys. Rev. B* **94**, 045436 (2016).

82. T. Tang, W. Ding, W. Fu, S. Tang, X. Zhang, Scale-dependent anomalous behavior of confined water between Al_2O_3 layers. *Nano Res.* **18**, 94907417 (2025).

83. X. Chen, Y. Qin, Y. Zhu, X. Pan, Y. Wang, H. Ma, R. Wang, C. D. Easton, Y. Chen, C. Tang, A. Du, A. Huang, Z. Xie, X. Zhang, G. P. Simon, M. M. Banaszak Holl, X. Lu, K. Novoselov, H. Wang, Accurate prediction of solvent flux in sub–1-nm slit-pore nanosheet membranes. *Sci. Adv.* **10**, eadl1455 (2024).

84. T. Chu, Z. Zhou, P. Tian, T. Yu, C. Lian, B. Zhang, F.-Z. Xuan, Nanofluidic sensing inspired by the anomalous water dynamics in electrical angstrom-scale channels. *Nat. Commun.* **15**, 7329 (2024).

85. S. Fleischmann, Y. Zhang, X. Wang, P. T. Cummings, J. Wu, P. Simon, Y. Gogotsi, V. Presser, V. Augustyn, Continuous transition from double-layer to Faradaic charge storage in confined electrolytes. *Nat. Energy* **7**, 222–228 (2022).

86. J. H. Park, N. R. Aluru, Ordering-induced fast diffusion of nanoscale water film on graphene. *J. Phys. Chem. C* **114**, 2595–2599 (2010).

87. N. Wei, X. Peng, Z. Xu, Breakdown of fast water transport in graphene oxides. *Phys. Rev. E* **89**, 012113 (2014).

88. K. G. Zhou, K. S. Vasu, C. T. Cherian, M. Neek-Amal, J. C. Zhang, H. Ghorbanfekr-Kalashami, K. Huang, O. P. Marshall, V. G. Kravets, J. Abraham, Y. Su, A. N. Grigorenko, A. Pratt, A. K. Geim, F. M. Peeters, K. S. Novoselov, R. R. Nair, Electrically controlled water permeation through graphene oxide membranes. *Nature* **559**, 236–240 (2018).

89. J. Kou, J. Yao, H. Lu, B. Zhang, A. Li, Z. Sun, J. Zhang, Y. Fang, F. Wu, J. Fan, Electromanipulating water flow in nanochannels. *Angew. Chem. Int. Ed. Engl.* **54**, 2351–2355 (2015).

90. G. Kresse, J. Furthmüller, Efficient iterative schemes for ab initio total-energy calculations using a plane-wave basis set. *Phys. Rev. B* **54**, 11169–11186 (1996).

91. J. P. Perdew, K. Burke, M. Ernzerhof, Generalized gradient approximation made simple. *Phys. Rev. Lett.* **77**, 3865–3868 (1996).

92. S. Grimme, J. Antony, S. Ehrlich, H. Krieg, A consistent and accurate ab initio parametrization of density functional dispersion correction (DFT-D) for the 94 elements H-Pu. *J. Chem. Phys.* **132**, 154104 (2010).

93. P. E. Blöchl, Projector augmented-wave method. *Phys. Rev. B* **50**, 17953–17979 (1994).

94. G. Kresse, D. Joubert, From ultrasoft pseudopotentials to the projector augmented-wave method. *Phys. Rev. B* **59**, 1758–1775 (1999).

95. R. Jinnouchi, J. Lahnsteiner, F. Karsai, G. Kresse, M. Bokdam, Phase transitions of hybrid perovskites simulated by machine-learning force fields trained on the fly with bayesian inference. *Phys. Rev. Lett.* **122**, 225701 (2019).

96. R. Jinnouchi, K. Miwa, F. Karsai, G. Kresse, R. Asahi, On-the-fly active learning of interatomic potentials for large-scale atomistic simulations. *J. Phys. Chem. Lett.* **11**, 6946–6955 (2020).

97. C. Verdi, F. Karsai, P. Liu, R. Jinnouchi, G. Kresse, Thermal transport and phase transitions of zirconia by on-the-fly machine-learned interatomic potentials. *npj Comput. Mater.* **7**, 156 (2021).

98. B. W. J. Chen, X. Zhang, J. Zhang, Accelerating explicit solvent models of heterogeneous catalysts with machine learning interatomic potentials. *Chem. Sci.* **14**, 8338–8354 (2023).

99. S. Nosé, A unified formulation of the constant temperature molecular dynamics methods. *J. Chem. Phys.* **81**, 511–519 (1984).

100. S. Nosé, Constant temperature molecular dynamics methods. *Prog. Theor. Phys. Suppl.* **103**, 1–46 (1991).

101. G. E. Granroth, A. I. Kolesnikov, T. E. Sherline, J. P. Clancy, K. A. Ross, J. P. C. Ruff, B. D. Gaulin, S. E. Nagler, Sequoia: A newly operating chopper spectrometer at the SNS. *J. Phys. Conf. Ser.* **251**, 012058 (2010).

102. M. B. Stone, J. L. Niedziela, D. L. Abernathy, L. DeBeer-Schmitt, G. Ehlers, O. Garlea, G. E. Granroth, M. Graves-Brook, A. I. Kolesnikov, A. Podlesnyak, B. Winn, A comparison of four direct geometry time-of-flight spectrometers at the Spallation Neutron Source. *Rev. Sci. Instrum.* **85**, 045113 (2014).

103. M. A. Caro, T. Laurila, O. Lopez-Acevedo, Accurate schemes for calculation of thermodynamic properties of liquid mixtures from molecular dynamics simulations. *J. Chem. Phys.* **145**, 244504 (2016).

104. S.-T. Lin, M. Blanco, W. A. Goddard, III, The two-phase model for calculating thermodynamic properties of liquids from molecular dynamics: Validation for the phase diagram of Lennard-Jones fluids. *J. Chem. Phys.* **119**, 11792–11805 (2003).

105. S.-T. Lin, P. K. Maiti, W. A. Goddard, III, Two-phase thermodynamic model for efficient and accurate absolute entropy of water from molecular dynamics simulations. *J. Phys. Chem. B* **114**, 8191–8198 (2010).

106. T. A. Pascal, S.-T. Lin, W. A. Goddard, III, Thermodynamics of liquids: Standard molar entropies and heat capacities of common solvents from 2PT molecular dynamics. *Phys. Chem. Chem. Phys.* **13**, 169–181 (2011).

107. W. Xu, Z. Shi, Z. Yu, C. Peng, G. Yang, H.-F. Wang, J. Huang, Y. Cao, H. Wang, L. Li, H. Yu, A sweet synthesis of MXenes. *Nano Lett.* **24**, 10547–10553 (2024).

108. Z. Jin, C. Liu, Z. Liu, J. Han, Y. Fang, Y. Han, Y. Niu, Y. Wu, C. Sun, Y. Xu, Rational design of hydroxyl-rich $Ti_3C_2T_x$ MXene quantum dots for high-performance electrochemical N_2 reduction. *Adv. Energy Mater.* **10**, 2000797 (2020).

109. M. Schied, H. Pazniak, F. Brette, P. Lacovig, M. Paris, F. Boucher, S. Lizzit, V. Mauchamp, R. Larciprete, Reactivity of $Ti_3C_2T_x$ MXene with atomic hydrogen: Tuning of surface terminations by halogen removal and reversible O to OH conversion. *Chem. Mater.* **36**, 11905–11919 (2024).

110. E. Mamontov, K. W. Herwig, A time-of-flight backscattering spectrometer at the Spallation Neutron Source, BASIS. *Rev. Sci. Instrum.* **82**, 085109 (2011).

111. A. Meyer, R. M. Dimeo, P. M. Gehring, D. A. Neumann, The high-flux backscattering spectrometer at the NIST Center for Neutron Research. *Rev. Sci. Instrum.* **74**, 2759–2777 (2003).

112. Q. Berrod, K. Lagrené, J. Ollivier, J.-M. Zanotti, Inelastic and quasi-elastic neutron scattering. Application to soft-matter. *EPJ Web Conf.* **188**, 05001 (2018).

113. N. C. Osti, E. Mamontov, Microscopic dynamics in room-temperature ionic liquids confined in materials for supercapacitor applications. *Sustain. Energ. Fuels* **4**, 1554–1576 (2020).

114. H.-W. Wang, M. Naguib, K. Page, D. J. Wesolowski, Y. Gogotsi, Resolving the structure of $Ti_3C_2T_x$ MXenes through multilevel structural modeling of the atomic pair distribution function. *Chem. Mater.* **28**, 349–359 (2016).

115. E. S. Muckley, M. Naguib, H.-W. Wang, L. Vlcek, N. C. Osti, R. L. Sacci, X. Sang, R. R. Unocic, Y. Xie, M. Tyagi, E. Mamontov, K. L. Page, P. R. C. Kent, J. Nanda, I. N. Ivanov,

Multimodality of structural, electrical, and gravimetric responses of intercalated MXenes to water. *ACS Nano* **11**, 11118–11126 (2017).

116. Y. Ying, Y. Liu, X. Wang, Y. Mao, W. Cao, P. Hu, X. Peng, Two-dimensional titanium carbide for efficiently reductive removal of highly toxic chromium(VI) from water. *ACS Appl. Mater. Interfaces* **7**, 1795–1803 (2015).

117. M. Khazaei, M. Arai, T. Sasaki, C.-Y. Chung, N. S. Venkataramanan, M. Estili, Y. Sakka, Y. Kawazoe, Novel electronic and magnetic properties of two-dimensional transition metal carbides and nitrides. *Adv. Funct. Mater.* **23**, 2185–2192 (2013).

118. M. Ashton, K. Mathew, R. G. Hennig, S. B. Sinnoet, Predicted surface composition and thermodynamic stability of MXenes in solution. *J. Phys. Chem. C* **120**, 3550–3556 (2016).

119. G. Herzberg, A. Monfils, The dissociation energies of the H_2, HD, and D_2 molecules. *J. Mol. Spectrosc.* **5**, 482–498 (1961).

120. V. I. Vedeneyev, *Bond Energies, Ionization Potentials and Electron Affinities* (St. Martin's Press, 1966).

121. J. W. C. Johns, R. F. Barrow, C. N. Hinshelwood, The ultra-violet spectra of HF and DF. *Proc. R. Soc. Lond. A Math. Phys. Sci.* **251**, 504–518 (1997).
